# Red-emissive azabenzanthrone derivatives for photodynamic therapy irradiated with ultralow light power density and two-photon imaging†
†Electronic supplementary information (ESI) available: Experimental details for the synthesis, ^1^H/^13^C NMR spectra, IR spectra, HRMS spectra, and absorption and emission spectra of **1a–b** and **2a–b**, the single crystal data of **1a** and **1b** (CCDC numbers: 1822247 and 1822250), the preparation of nanoparticles, the ROS generation measurements, the determination of ^1^O_2_ quantum yield, cell culture and bio-imaging experiments, the measurement of the two-photon absorption cross section, cytotoxicity studies and cell staining. For ESI and crystallographic data in CIF or other electronic format see DOI: 10.1039/c8sc00633d


**DOI:** 10.1039/c8sc00633d

**Published:** 2018-04-24

**Authors:** Qiguang Zang, Jiayi Yu, Wenbin Yu, Jun Qian, Rongrong Hu, Ben Zhong Tang

**Affiliations:** a State Key Laboratory of Luminescent Materials and Devices , Center for Aggregation-Induced Emission , South China University of Technology , Guangzhou 510640 , China . Email: msrrhu@scut.edu.cn ; Email: tangbenz@ust.hk; b State Key Laboratory of Modern Optical Instrumentation , Centre for Optical and Electromagnetic Research , Zhejiang Provincial Key Laboratory for Sensing Technologies , Zhejiang University , Hangzhou , China; c Department of Chemistry , Hong Kong Branch of Chinese National Engineering Research Center for Tissue Restoration and Reconstruction , The Hong Kong University of Science & Technology , Clear Water Bay , Kowloon , Hong Kong , China

## Abstract

Photodynamic therapy has proved to be an effective strategy for cancer therapy, and advanced photosensitizers for image-guided photodynamic therapy require biocompatibility, intense absorption, high ROS generation efficiency, phototoxicity, low irradiation power density and efficient emission. In this work, four red emissive azabenzanthrone derivatives have been designed and synthesized, which generally exhibit efficient aggregated state emission. Through structural optimization, 3-diphenylamino-11-azabenzanthrone was found to show satisfactory photo-induced ROS generation and high emission efficiency in the aggregated state. Under the irradiation of a white LED lamp with an ultralow power density of 1.67 mW cm^–2^, this compound demonstrates significant photo-induced cytotoxicity toward HeLa cells. Moreover, deep tissue penetration can be realized by two-photon imaging of mouse brain vessels with these azabenzanthrone derivatives at vertical depths of up to 280 μm, attributed to the large emission wavelength and efficient emission.

## Abstract 2

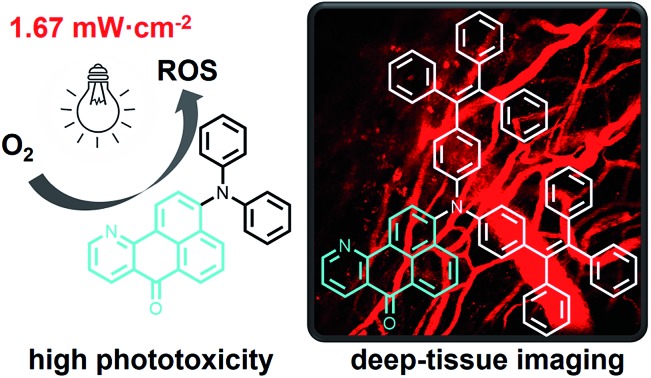
Azabenzanthrone derivatives with high phototoxicity and efficient emission are designed for photodynamic therapy and deep-tissue imaging.

## Introduction

Photodynamic therapy (PDT), using photosensitizers to generate toxic reactive oxygen species (ROS) and working as a light-sensitive drug to treat the target tissue locally upon exposure to light and kill tumour cells, has proved to be an effective strategy for cancer therapy.^1^,^2^ Compared with conventional cancer therapies, such as surgery, chemotherapy, and radiotherapy, PDT enjoys a series of advantages such as its non-invasive nature, high spatiotemporal precision, fewer side effects, less damage to marginal tissues, and fast healing of healthy tissue.^3^,^4^ An effective photosensitizer for PDT should possess biocompatibility, intense absorption and high ROS generation efficiency. Besides, low cytotoxicity in the absence of light and high cytotoxicity in the presence of light are generally required for excellent photosensitizers.^5^ For image-guided PDT, which enables real time fluorescence monitoring of the target site where therapy can be precisely applied by selective irradiation with a beam of light,^6^,^7^ photosensitizers with both efficient ROS generation and emission are required.^8^ Traditional photosensitizers such as porphyrins, methylene blue, and BODIPYs generally suffer from the formation of aggregates,^9^ leading to quenched fluorescence,^10^ dramatic reduction in ROS generation,^11^ reduced phototoxicity,^12^ and depressed imaging quality and PDT performance.^13^

The light sources used in PDT to irradiate photosensitizers for ROS generation are generally a laser beam or light with power density larger than 100 mW cm^–2^.^14^,^15^ However, the high irradiation intensity or long irradiation time generally used for efficient PDT may damage normal tissue around the therapeutic site by the photothermal effect and limit the effective therapeutic depth.^5^ Recently, red-emissive organic nanoparticles have been reported to offer deep-tissue photodynamic therapy using a low-power-density and cost-effective lamp light (12 mW cm^–2^).^16^–^18^ To realize therapy and simultaneous imaging at large depths, the development of new photosensitizers which require low irradiation power density and efficient ROS generation and emission is in great demand.

Aggregation-induced emission (AIE) compounds with efficient emission in the aggregated state or as nanoparticles in aqueous media have recently proved to be promising bio-probes.^19^,^20^ Unlike other photosensitizers whose nanoparticles generally possess poor emission due to the aggregation-caused emission quenching effect, AIE dyes exhibit both efficient emission and high phototoxicity in the aggregated state under physiological conditions,^21^ enabling wide application in image-guided therapy,^22^ drug delivery,^23^ and tumour diagnosis.^24^ Among them, red/NIR emissive AIE dyes with large conjugation and strong electron donor/acceptor structures enjoy advantages such as deep tissue penetration,^25^–^27^ and low biological autofluorescence interference.^28^ In fact, red emissive or NIR emissive small-molecule fluorescent probes have recently become ideal candidates as two-photon probes for bio-imaging of zebrafish embryos and mouse tissues.^29^–^34^

In this work, four red emissive compounds, which generally possess satisfactory emission quantum efficiency in the aggregated state, containing a unique azabenzanthrone electron acceptor are designed and synthesized. These compounds can be internalized in the lysosome of cells which demonstrate high photostability during the imaging process. Most importantly, upon irradiation at a low power density of 2.0 J cm^–2^, cell viability decreased dramatically to 4% after treatment with 7.5 mM dye. Two-photon imaging of mouse brain vessels with the azabenzanthrone derivative can also reach a depth of 280 μm with excitation at 1040 nm.

## Results and discussion

### Synthesis and characterization

A coplanar azabenzanthrone core was designed and synthesized as a new electron acceptor. Under the catalysis of ZnCl_2_, **5** was obtained from the reaction of **3** and **4** and further underwent amination to afford precursors **6** and **6′**, which was then treated with glycol and H_2_SO_4_ to furnish a pair of isomers **7** and **8** with bromo atoms located at the 3- or 4-position of the azabenzanthrone core respectively, enabling further modification.^35^,^36^ The coupling reaction of the mixture of **7** and **8** with diphenylamine catalyzed by Pd(OAc)_2_ produces isomers **1a** and **1b** with different polarities, which can be isolated by chromatography. Similarly, compounds **2a** and **2b** were synthesized and isolated (Scheme S1†).

**Scheme 1:**
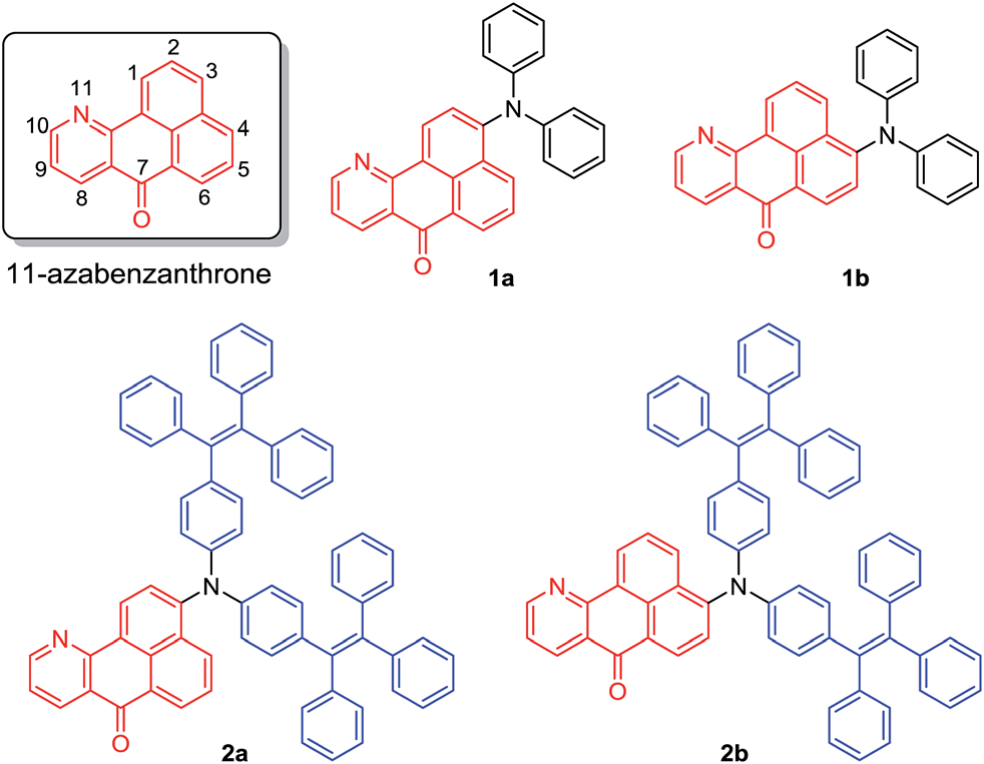
Chemical structures of 11-azabenzanthrone derivatives **1a–b** and **2a–b**.

The structures of **1a–b** and **2a–b** are fully characterized with satisfactory analysis results shown in the ESI.† For example, their ^1^H NMR spectra suggest that multiple peaks emerged at low field at *δ* 9.13–7.32, owing to the deshielding effect of the coplanar structure, proving the existence of the azabenzanthrone core structure (Fig. S1†). Their ^13^C NMR spectra all possess carbonyl peaks located at *δ* 182.71–183.87 (Fig. S2†), which was also confirmed by their IR spectra with the carbonyl peaks located at 1645–1655 cm^–1^ (Fig. S3†). Their high resolution-mass spectra gave M^+^ peaks at *m*/*z* 398.1429 (calcd for **1a**, 398.1414), 398.1425 (calcd for **1b**, 398.1414), 906.3590 (calcd for **2a**, 906.3605), and 906.3617 (calcd for **2b**, 906.3605), respectively (Fig. S4–S7†), confirming the expected structures of azabenzanthrone derivatives shown in Scheme 1. Moreover, single crystal structures of **1a–b** were obtained to confirm their expected structures and reveal their molecular conformations (Table S1†).

### Photophysical properties

The photophysical properties of the azabenzanthrone derivatives were investigated. The UV-vis absorption spectra of THF solutions of **1a–b** and **2a–b** were recorded and their absorption maxima were found at 486 nm (**1a**), 469 nm (**1b**), 515 nm (**2a**), and 499 nm (**2b**), with their molar absorptivity up to 29 400 L mol^–1^ cm^–1^ (Fig. S8†), indicating better conjugation of **1a** and **2a** compared with **1b** and **2b**. With their strong D–A structures, the solvatochromic effect of **1a** and **1b** on absorption and emission was studied in various solvents with different polarities (Fig. S9 and S10, Table S2†). When the solvent was changed from nonpolar hexane to polar methanol, the absorption maximum showed a slight redshift of about 15 nm. On the other hand, the emission spectra were highly dependent on the solvent polarity. For example, the emission maximum of **1a** in nonpolar hexane located at 529 nm was bathochromically shifted to 662 nm in DMSO, demonstrating a strong solvatochromic effect. Similarly, the emission maximum of **1b** redshifted by about 100 nm upon changing from nonpolar solvent to polar DMSO. Furthermore, the fluorescence quantum efficiency of **1a** in hexane (83%) gradually decreased with increasing solvent polarity, demonstrating its twisted intramolecular charge transfer (TICT) characteristics.

**Fig. 1:**
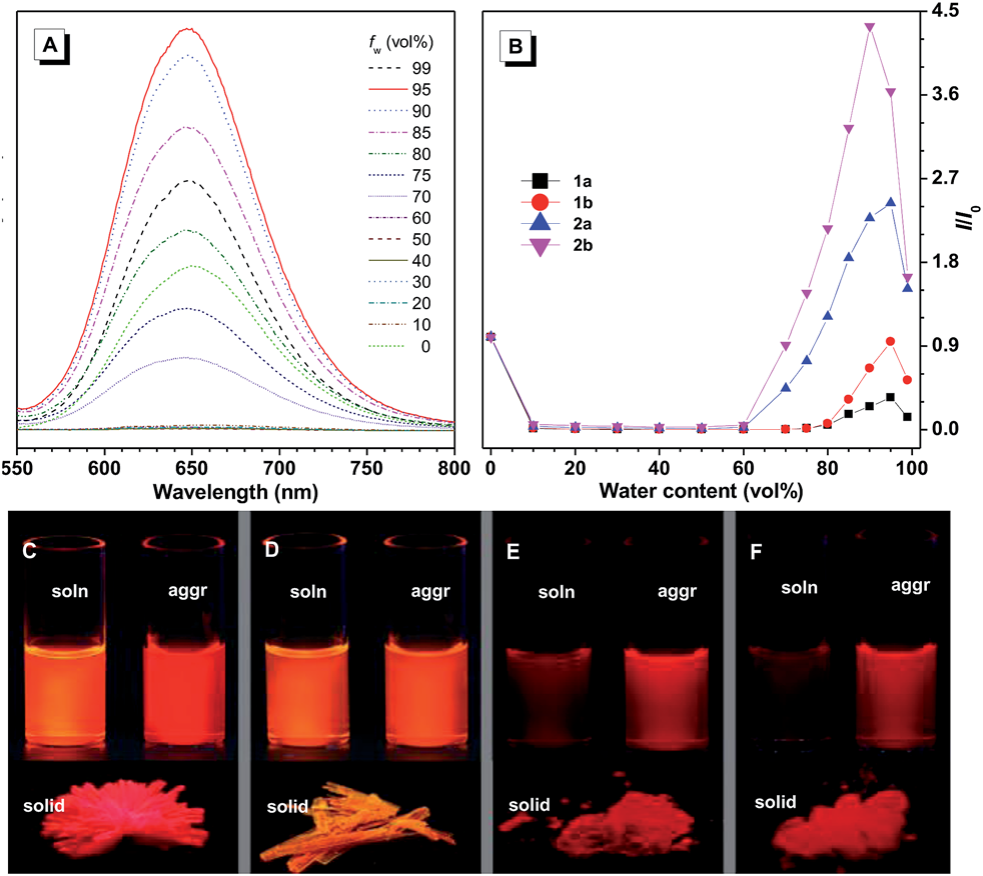
(A) PL spectra of **2a** in THF/water mixtures with different water contents (0–99 vol%). (B) Plots of relative PL intensity (*I*/*I*_0_) of **1a–b** and **2a–b**, where *I*_0_ is the PL intensity of the THF solutions. *λ*_ex_ = 485 nm (**1a**), 470 nm (**1b**), 515 nm (**2a**), and 499 nm (**2b**). Concentration: 10 μM. Fluorescence photographs of THF solutions, nanoaggregates in THF/water mixtures with 99 vol% water, and solid powders of (C) **1a**, (D) **1b**, (E) **2a**, and (F) **2b** taken under 365 nm UV irradiation.

Their emission spectra were recorded in THF solutions, aggregated states in aqueous media, and as solid powders, respectively (Fig. S11† and Table 1). From solution to solid state, the emission maximum of **1a** red-shifted by 28 nm, while the other three compounds did not show any obvious change, indicating different intermolecular interactions. The emission maxima of the solid powders of these compounds are located at 635 nm (**1a**), 599 nm (**1b**), 659 nm (**2a**), and 652 nm (**2b**), generally emitting red light. Meanwhile, the fluorescence quantum efficiencies of **1a–b** decreased from solution to solid state. In contrast, those of **2a–b** increased from solution to solid state. The solutions of **2a–b** are less emissive compared with those of **1a–b**, due to the large number of intramolecular rotatable phenyl rings which consume excited state energy by non-radiative decay. Their emission spectra were also recorded in THF/water mixtures with different amounts of water, which serves as a poor solvent. For example, the THF solution of **2a** emits faintly, and when a small amount of water (<60 vol%) is added into the solution, the fluorescence is further quenched, which can be attributed to the polarity change of the solvent. The TICT process might take place to decrease the emission intensity in such mixed polar solvents. When the water content was further increased above 70 vol%, remarkable fluorescence was observed at about 649 nm, the intensity of which was further increased upon addition of water (Fig. 1), demonstrating typical aggregation-induced emission characteristics. A similar phenomenon can be observed for the other three compounds, that is, their emission intensity first decreased and then increased upon addition of water in aqueous solution (Fig. S12†).

**Table 1:** Photophysical properties of **1a–b** and **2a–b**^*a*^

Compd	*λ*_ab_ (nm)	*ε* (L mol^–1^ cm^–1^)	*λ*_em_ ^*b*^ (nm)			*Φ* ^*b*^ (%)			*τ* ^*c*^ (ns)		
			Soln	Aggr	Solid	Soln	Aggr	Solid	Soln	Aggr	Solid
**1a**	486	21 300	607	618	635	38.6	5.3	22.8	9.83	2.66	12.15
**1b**	469	26 900	598	601	599	20.7	13.0	6.1	7.10	6.02	4.36
**2a**	515	22 600	655	649	659	4.0	8.2	11.6	1.60	2.10	3.11
**2b**	499	29 400	648	635	652	2.4	3.7	8.0	1.04	1.88	2.74

^*a*^Abbreviations: *λ*_ab_ = absorption maximum in THF solution (10 μM), *λ*_em_ = emission maxima in THF solutions (10 μM), nanoaggregates in THF/water mixtures with 99 vol% water content (10 μM), and solid powders, *Φ* = fluorescence quantum yields determined by a calibrated integrating sphere, and *τ* = fluorescence lifetime.

^*b*^ *λ*_ex_ = *λ*_ab_.

^*c*^ *λ*_ex_ = 470 nm.

When the diphenylamine electron donor is located at the 3-position of azabenzanthrone in **1a**, the D–A effect is stronger compared with that of **1b** where the electron donor is substituted at the 4-position of azabenzanthrone, leading to a stronger solvatochromic effect of **1a** than **1b**. A similar trend is observed for **2a** and **2b**, that is, the electron donor located at the 3-position produces a stronger D–A effect. Meanwhile, **2a–b** possess red-shifted absorption and emission compared with **1a–b**, owing to the extended conjugation from diphenylamine to di(tetraphenylethene)amine groups.

**Fig. 2:**
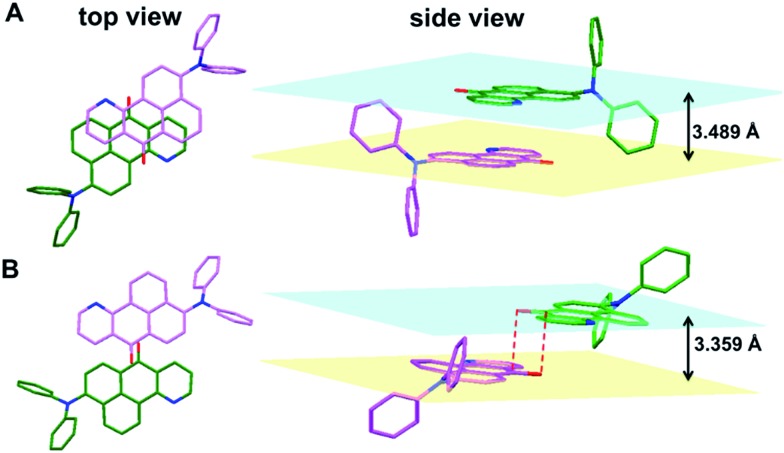
The single crystal structures and molecular packing structures of (A) **1a** and (B) **1b**.

Intermolecular interactions also play an important role in the emission of their aggregated states or solid states. The single crystal X-ray diffraction measurements of **1a–b** indicate that in the single crystal structure of **1a**, two azabenzanthrone planes adopt an antiparallel alignment with an interplanar distance of 3.489 Å, and a large overlap between two azabenzanthrone planes can be observed from the top view (Fig. 2). In contrast, in the single crystal structure of **1b**, although the azabenzanthrone core also adopts an antiparallel alignment with an interplanar distance of 3.359 Å, the overlap is barely observed, suggesting weak intermolecular interactions. The strong intermolecular stacking of **1a** is also responsible for the redshifted emission maximum and higher emission efficiency in the solid state compared with that of **1b**, owing to the restricted intramolecular motions.

### Photodynamic therapy and bio-imaging

**Fig. 3:**
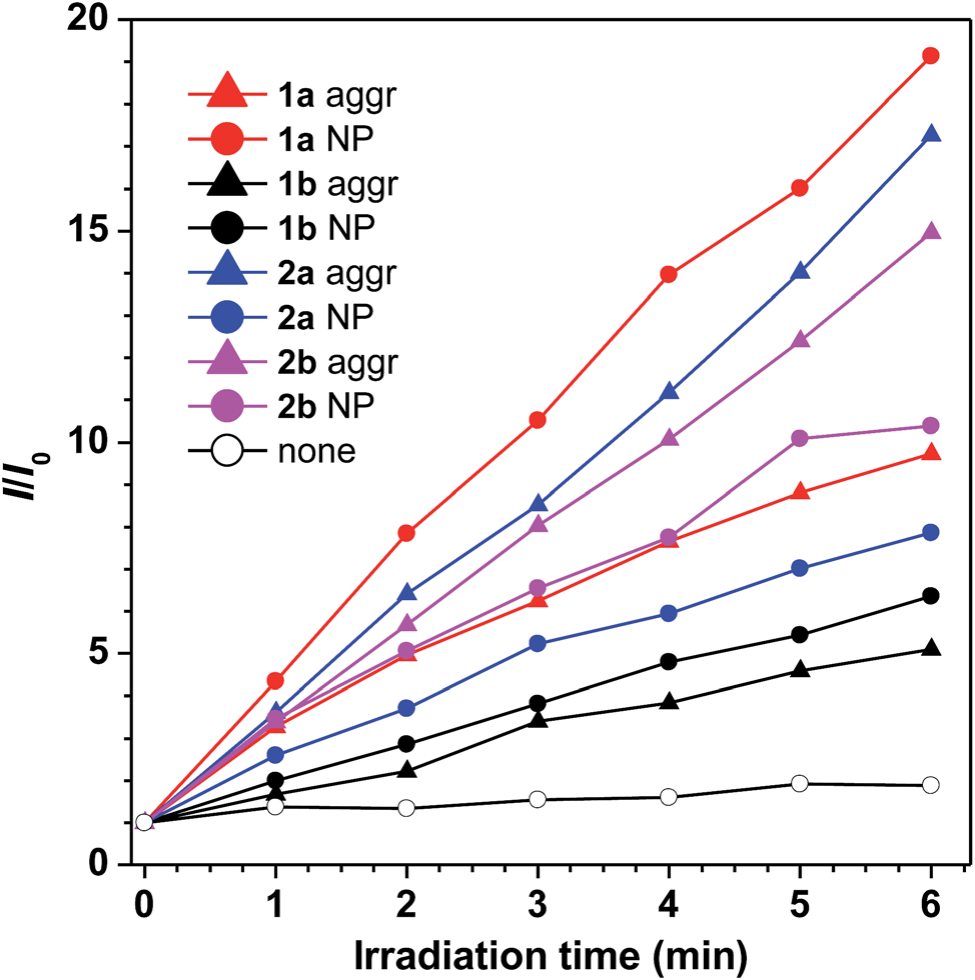
Plots of relative fluorescence intensity (*I*/*I*_0_) of the nanoaggregates in THF/water mixtures with 99 vol% water contents and the nanoparticles of **1a–b** and **2a–b***versus* different light irradiation times. Concentration: 10 μM, *λ*_em_ = 525 nm, irradiation light: 1.5 mW cm^–2^ white visible light from a LED lamp.

For **1a–b** and **2a–b**, nanoaggregates dispersed in aqueous suspensions and nanoparticles encapsulated in DSPE-PEG_2000_-OCH_3_ were prepared with their particle size ranging from 120 to 170 nm (Table S3†). Their photo-induced ROS producing efficiency was evaluated with the ROS probe DCFH-DA. Even after irradiation with a LED lamp with 1.5 mW cm^–2^ white visible light for 6 min, the PL intensity of the blank solution at 525 nm was barely increased. However, in the presence of the aqueous suspension or nanoparticles of **1a** with a concentration of 1.0 × 10^–5^ M, the PL intensity at 525 nm increased sharply upon white light irradiation (Fig. 3). Other compounds also show a similar response upon white light irradiation and their ^1^O_2_ quantum yields were measured using Rose Bengal as the standard photosensitizer and SOSG as the probe (Fig. S13†). Among them, **1a** with the highest solid state fluorescence efficiency of 22.8% possesses the highest ^1^O_2_ quantum yield of 12.9% in its nanoaggregate form, indicating its potential application in photodynamic therapy.

**Fig. 4:**
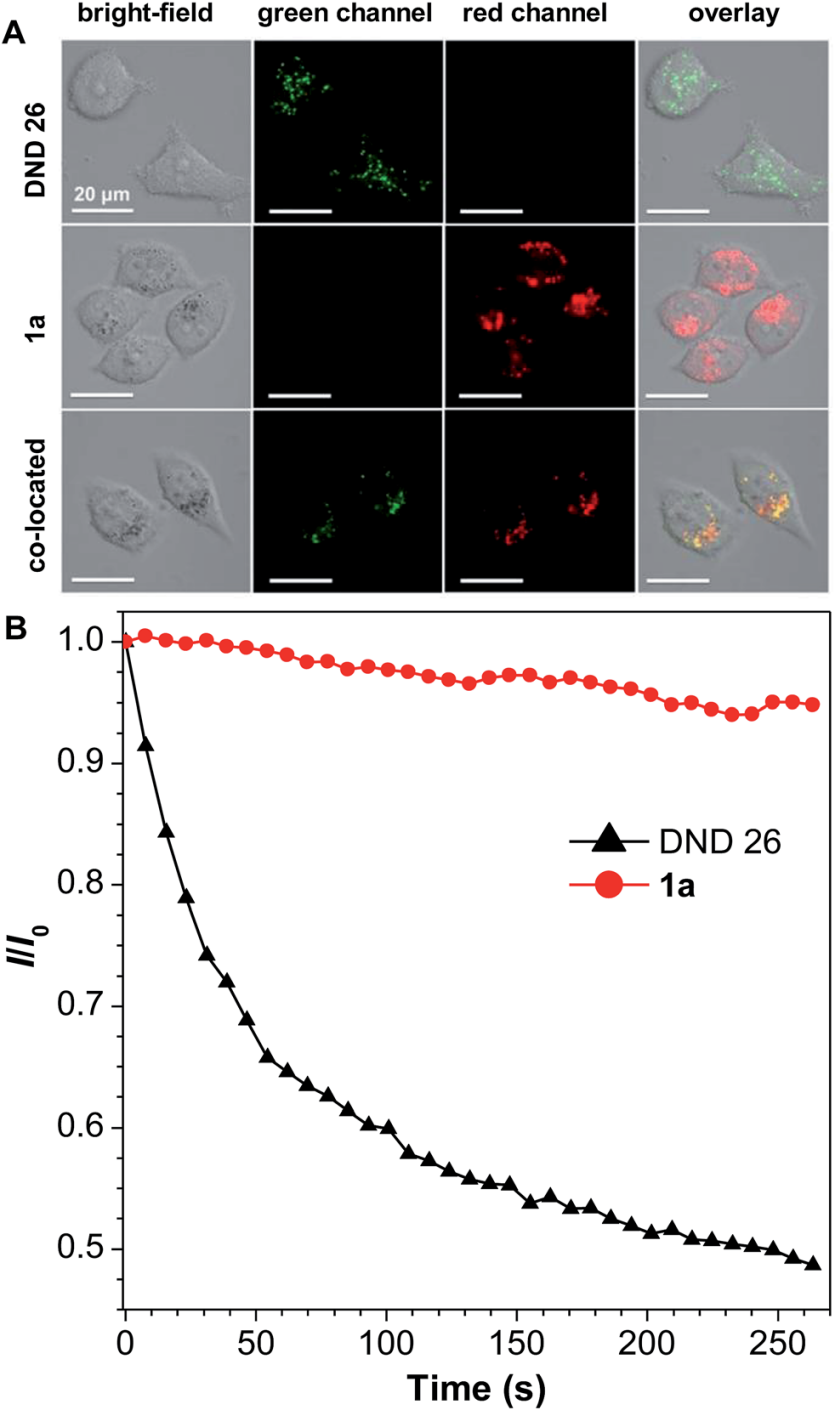
(A) Confocal images of HeLa cells after incubation with **1a** (5 μM) and/or LysoTracker DND-26 (0.5 μM) for 2 h observed in the bright field, the green channel (500–540 nm), the red channel (620–740 nm), and the overlap of the green and red channels. (B) The relative fluorescence intensity (*I*/*I*_0_) of **1a** and LysoTracker DND-26 *versus* different light irradiation times. *λ*_ex_ = 488 nm.

**1a** was then selected to be incubated with HeLa cells and co-stained with LysoTracker DND-26 for comparison. A confocal laser scanning microscope was used to image the fluorescence signal of **1a** at 620–740 nm and LysoTracker DND-26 at 500–540 nm. After 2 h of incubation, it was found that the molecules of **1a** could enter the cell and were mainly localized in lysosomes (Fig. 4A), indicating good biocompatibility. The photostability of **1a** in living HeLa cells was then evaluated using a 488 nm laser to scan the same field continuously, and the fluorescence intensity was recorded. After exposure for 270 s, the fluorescence intensity of LysoTracker DND-26 was reduced to less than 50% of its initial value, while larger than 95% of the fluorescence intensity of **1a** was retained under the same conditions, proving its excellent photostability (Fig. 4B).

**Fig. 5:**
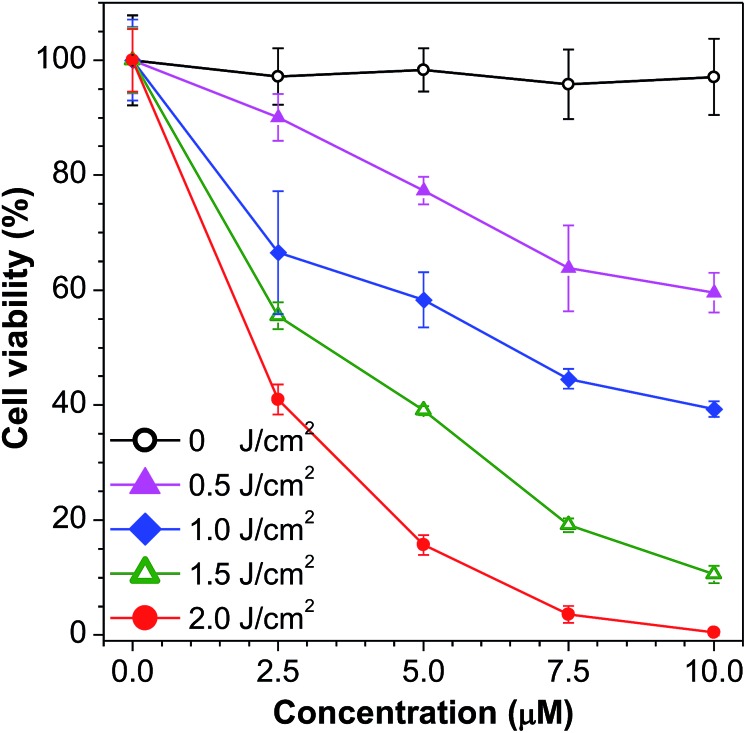
Cell viability of HeLa cells after incubation with **1a** for 2 h and exposure to light from a white LED lamp (1.67 mW cm^–2^) for 0, 5, 10, 15, and 20 min.

To investigate the cytotoxicity of **1a***in vivo* with and without white light irradiation, an MTT method was used to study the cell viability of HeLa cells (Fig. 5). Without light irradiation, the cytotoxicity of **1a** is proved to be negligible, which remained above 95% after HeLa cells were treated with 1.5 × 10^–5^ M of **1a** for 24 h (Fig. S14†). However, when the white LED light was turned on, even with an ultralow power density of 1.67 mW cm^–2^, cell viability decreased dramatically. The cells were killed almost completely when 2.0 J cm^–2^ white light was used to irradiate the cells with 1.0 × 10^–5^ M of **1a**, indicating high photo-induced cytotoxicity. The cell imaging of HeLa cells incubated with **1a** with exposure to increasing irradiation energy reveals a gradual increase in the proportion of dead cells stained by PI and a gradual decrease in the proportion of living cells stained by fluorescein (Fig. S15†).

**Fig. 6:**
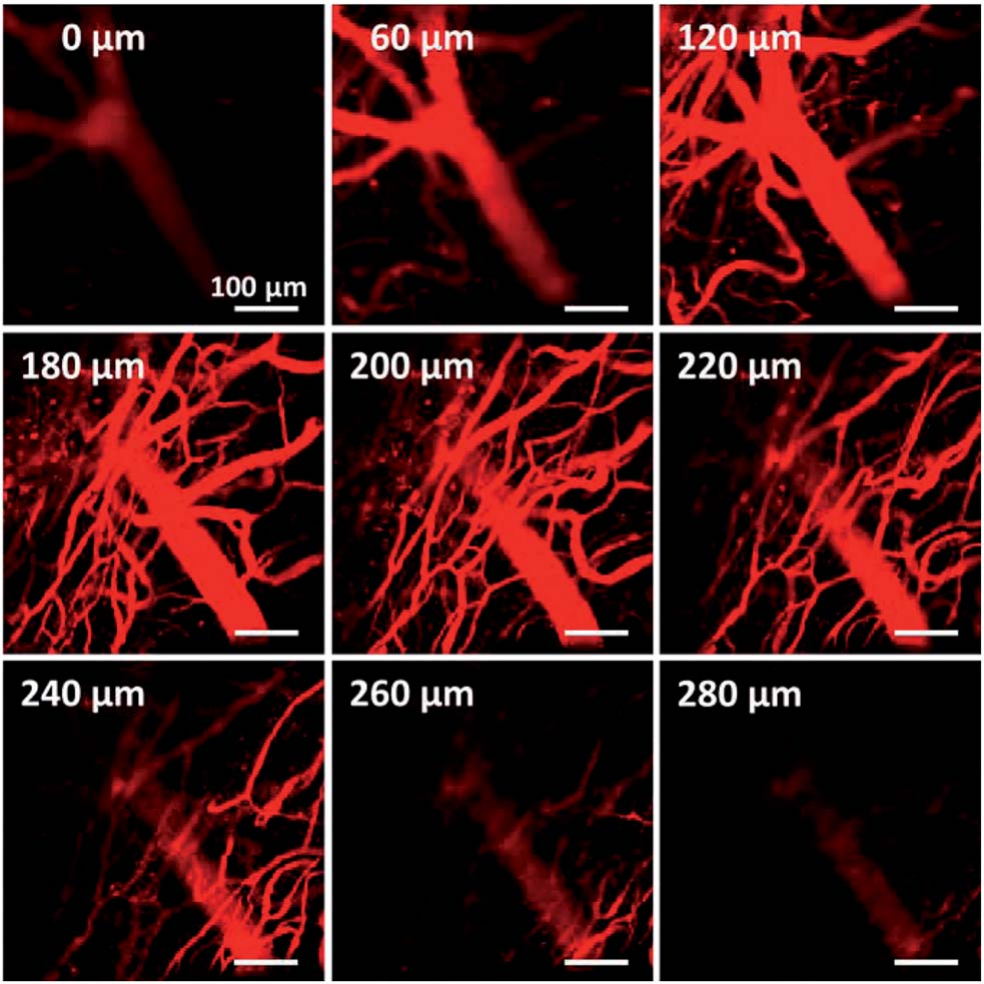
Two-photon fluorescence images of mouse brain vessels stained with nanoparticles of **2a** at different vertical depths. The fluorescence signal is collected upon excitation at 1040 nm. The signal is collected in the 560–700 nm range. Scale bars: 100 μm.

The electron donor–acceptor structure and large conjugation of the azabenzanthrone derivatives might endow them with two-photon absorption properties. Two-photon imaging of mouse brain vessels with nanoparticles of **2a** was then conducted as an example using a 1040 nm laser as the excitation light. Clear vessels can be imaged at different vertical depths, and fluorescence signals can be observed at depths of up to 280 μm, suggesting the potential for deep tissue penetration (Fig. 6).

## Conclusions

In conclusion, a series of new photosensitizers, azabenzanthrone derivatives, have been designed and synthesized. The structure–property relationship regarding their photophysical properties reveals that when an electron donor group is substituted at the 3-position, the compounds generally possess redshifted absorption and emission, higher solid state fluorescence quantum efficiency, and a stronger solvatochromic effect compared with the 4-substituted azabenzanthrone derivatives. Among these compounds, 3-diphenylamino-11-azabenzanthrone shows satisfactory photo-induced ROS generation and high emission efficiency in its aggregated state. It is demonstrated that this compound exhibits high phototoxicity toward HeLa cells even under irradiation with a cost-effective ultralow power LED lamp. Deep tissue penetration can be realized by two-photon imaging of mouse brain vessels with these azabenzanthrone derivatives. The high photo-induced cytotoxicity and efficient emission in the aggregated state suggest the potential application of the azabenzanthrone derivatives for non-invasive image-guided photodynamic therapy.

## Experimental section

### Materials and instruments

Fluorescein diacetate, 4-bromonaphthalic anhydride, NH_4_OAc, and diethyl malonate were purchased from Energy Chemical Ltd; ZnCl_2_, sodium *m*-nitrobenzenesulfonate (90%), diphenylamine, tri-*tert*-butylphosphine solution (0.1 mol L^–1^ in toluene), palladium acetate, glycerol, Cs_2_CO_3_, Rose Bengal (RB), and 3-(4,5-dimethyl-2-thiazolyl)-2,5-diphenyl-2*H*-tetrazolium bromide (MTT) were purchased from J&K Scientific Ltd; DSPE-PEG_2000_-OCH_3_ was purchased from Corden Pharma; toluene, *n*-hexane, ethyl acetate, THF, acetone, CH_2_Cl_2_, chloroform, DMSO, methanol, NaOH, H_2_SO_4_ (95–98 wt%), and anhydrous Na_2_SO_4_ were purchased from Guangzhou Chemical Reagent Factory; 2,7-dichlorodihydrofluorescein diacetate (DCFH-DA) and F127 were purchased from Sigma-Aldrich; ultrapure water was supplied by a Milli-Q Plus System from Millipore Corporation; phosphate buffered solution (PBS), Dulbecco's Modified Essential Medium (DMEM), fetal bovine serum (FBS), penicillin, streptomycin, propidium iodide (PI) and LysoTracker DND-26 were purchased from Thermo Fisher Scientific; Singlet Oxygen Sensor Green (SOSG) was purchased from Invitrogen; Rhodamine B (RhB) was purchased from Sigma-Aldrich; HeLa cells were obtained from the Cell Culture Center of the Institute of Basic Medical Sciences, Chinese Academy of Medical Science; 96-well plates were purchased from SARSTEDT; confocal imaging dishes were purchased from Thermo Scientific. All the commercially available reactants and reagents were used as received without further purification. 4-(1,2,2-Triphenylethenyl)-*N*-[4-(1,2,2-triphenylethenyl)phenyl]-benzenamine **10** was synthesized according to the literature.^30^

^1^H and ^13^C NMR spectra were measured on a Bruker Avance 500 NMR spectrometer using CDCl_3_ as the solvent and tetramethylsilane (TMS, *δ* = 0) as the internal reference. High resolution mass spectra (HRMS) were recorded on a GCT Premier CAB 048 mass spectrometer operated in MALDI-TOF mode. FT-IR spectra were recorded on a Bruker Vector 33 FT-IR spectrometer. UV-vis absorption and photoluminescence spectra were recorded on a SHIMADZU UV-2600 spectrophotometer and HORIBA Fluoromax-4 spectrofluorometer, respectively. The absolute fluorescence quantum yields were measured on a Hamamatsu C11347 Absolute Quantum Yield Spectrometer. The time-resolved fluorescence spectra were measured on a Hamamatsu C11367 Compact Fluorescence Lifetime Spectrometer. Single crystal X-ray diffraction data were collected at 293 K on a Bruker-Nonius Smart Apex CCD diffractometer. The white light source used for ROS generation experiments and light cytotoxicity studies was a GUANDI GD-5W LED light source with an input power of 5 W. The irradiation light energy density was measured using an FZ-A irradiatometer produced by Beijing Normal University optoelectronic instrument factory. The ^1^O_2_ quantum yields were tested by using SOSG as the indicator after irradiation with a CXE-350 Xenon lamp from OPT Photoelectric Technology. Particle size analysis was performed on a Malvern Zetasizer Nano-S90. Confocal laser scanning microscopy (CLSM) images were obtained on a Zeiss LSM7 DUO Laser Scanning Confocal Microscope. The absorbance of MTT assays at 570 nm was recorded on a TECAN Infinite 200 PRO microplate reader. Two-photon blood vasculature images were obtained on an Olympus BX61+FV1200 two-photon fluorescence scanning microscope.

The detailed experimental section can be found in the ESI.†

## Conflicts of interest

There is no conflicts to declare.

## Supplementary Material

Supplementary information

Crystal structure data
