# Supplementary material for: Red-emissive azabenzanthrone derivatives for photodynamic therapy irradiated with ultralow light power density and two-photon imaging
Source: Chem Sci. 2018 Apr 24;9(23):5165–71. doi: 10.1039/c8sc00633d (PMC6000979; doi:10.1039/c8sc00633d)
Supplement: Supplementary file 1 [file SC-009-C8SC00633D-s001.pdf]

Electronic Supplementary Material (ESI) for Chemical Science.
This journal is © The Royal Society of Chemistry 2018

Electronic Supplementary Information

Red-emissive Azabenzanthrone Derivatives for Photodynamic Therapy Irradiated with Ultralow Light Power Density and Two-Photon Imaging

Qiguang Zang,[a] Jiayi Yu,[a] Jun Qian,[b] Wenbin Yu,[b] Rongrong Hu,[a] and Ben Zhong Tang[a,c]**

[a]State Key Laboratory of Luminescent Materials and Devices, Center for Aggregation-Induced Emission, South China University of Technology, Guangzhou 510640, China.
[b]State Key Laboratory of Modern Optical Instrumentations, Center for Optical and Electromagnetic Research, Zhejiang University, Hangzhou 310058, China.
[c]Department of Chemistry, Hong Kong Branch of Chinese National Engineering Research Center for Tissue Restoration and Reconstruction, The Hong Kong University of Science & Technology, Clear Water Bay, Kowloon, Hong Kong, China.
E-mail: msrrhu@scut.edu.cn, tangbenz@ust.hk

Table of Contents

Experimental details for the Synthesis and Characterizations of **1a-b** and **2a-b**, the preparation of nanoparticles, the ROS generation measurement, the determination of $^1O_2$ quantum yield, cell culture and bio-imaging experiments, the measurement of two-photon absorption cross section, cytotoxicity studies and cell staining.
Scheme S1. Synthesis routes of **1a-b** and **2a-b**.
Fig. S1 ^{1}H NMR spectra of (A) **1a**, (B) **1b**, (C) **2a**, and (D) **2b** in $CDCl_3$. The solvent peaks are marked with asterisks.
Fig. S2 ^{13}C NMR spectra of (A) **1a**, (B) **1b**, (C) **2a**, and (D) **2b** in $CDCl_3$.
Fig. S3 IR spectra of (A) **1a**, (B) **1b**, (C) **2a**, and (D) **2b**.
Fig. S4 HR-MS spectrum of **1a**.
Fig. S5 HR-MS spectrum of **1b**.
Fig. S6 HR-MS spectrum of **2a**.
Fig. S7 HR-MS spectrum of **2b**.
Table S1. Crystal data and structure refinement for single crystals **1a-b**.
Fig. S8 UV-Vis absorption spectra of THF solutions of **1a-b** and **2a-b**. Concentration: 10 μM.
Fig. S9 Normalized (A) UV-Vis absorption spectra and (B) PL spectra of **1a** in different solvents. Concentration: 10 μM.
Fig. S10 Normalized (A) UV-Vis absorption spectra and (B) PL spectra of **1b** in different solvents. Concentration: 10 μM.
Table S2. Photophysical properties of **1a-b** in different solvents.
Fig. S11 Normalized PL spectra of (A) **1a**, (B) **1b**, (C) **2a**, and (D) **2b** in solution,

aggregated and solid states. soln: in THF solution (10 μM); aggr: in THF/water mixture with 99 vol% water contents (10 μM); solid: solid powder of the compounds.

Fig. S12 PL spectra of (A) **1a**, (B) **1b**, and (C) **2b** in THF/water mixtures with different fractions of water (0-99 vol%), $\lambda_{ex} = \lambda_{ab}$, concentration: 10 μM.

Table S3. Particle size measurements of nanoaggregates and nanoparticles of **1a-b** and **2a-b** by dynamic light scattering technique

Fig. S13 The measurement of $^1O_2$ quantum yield of nanoaggregates and nanoparticles of **1a-b** and **2a-b** using SOSG as indicator.

Fig. S14 Cytotoxicity of **1a** for Hela cells without light irradiation. Cell viabilities of Hela cells were tested by MTT method.

Fig. S15 Cell imaging with fluorescein and PI for HeLa cells incubated with **1a** for 2 h in the dark and exposed to white light irradiation (1.67 $mW{\cdot}cm^{-2}$) with different irradiation energy. The living cells were stained by fluorescein diacetate (green, 500–525 nm channel, 50 μg/mL for 10 min) and the dead cells were stained by PI (red, 600–630 nm channel, 50 μg/mL for 10 min).

Synthesis and Characterizations:

Into a 250 mL two-necked round-bottom flask was added 4-Br-naphthalic anhydride **3** (5.55 g, 20 mmol), diethyl malonate **4** (18 mL, 110 mmol), and $ZnCl_2$ (5.0 g, 37 mmol). The reaction mixture was stirred at 145 °C for 5 hours, which was then stirred at 175 °C for 2 hours. After the solution mixture was cooled to room temperature, 50 mL of petroleum ether was added into the mixture to dissolve excess amount of diethyl malonate. The solvent was poured out and 50 g of NH_4OAc was added into the remaining black solid to react at 145 °C for 3 h. After the reaction mixture was cooled to room temperature, 500 mL of water was added to dissolve NH_4OAc. The crude products **6** and **6**' were obtained as a brown powder after filtration, which was dried overnight under vacuum to constant weight.

The crude products **6** and **6'** (1.0 g, 3.6 mmol) was mixed with sodium *m*-nitrobenzenesulfonate (2.2 g, 9 mmol), which was then added into 82 wt% H_2SO_4 (11 mL, 150 mmol). The solution was stirred at room temperature until the reactants were dissolved completely, glycerol (0.8 mL, 11 mmol) was added into the solution, which was stirred at 95 °C for 1 h, followed by reaction at 135 °C for 1.5 h. The reaction mixture was poured into 1 L of ice water which was extracted with dichloromethane for three times (50 mL × 3) and the organic phases were combined, which was dried with anhydrous Na_2SO_4. The solution was filtered and the solvent was removed under vacuum to obtain the crude product. The mixture of **7** and **8** were obtained in 19% as a yellow powder after isolation through silica column chromatography using dichloromethane as the eluent ($R_f = 0.65$).

Synthesis of 1a and 1b. Into a 100 mL two-necked round-bottom flask were added the mixture of **7** and **8** (0.31 g, 1.0 mmol), diphenylamine **9** (0.17 g, 1.0 mmol), Cs_2CO_3 (1.63 g, 5.0 mmol), and $Pd(OAc)_2$ (0.045 g, 0.2 mmol). The flask was vacuumed and purged with dry nitrogen for three times. After the solution of P(*t*-Bu)$_3$ (6.0 mL, 0.1

mol/L in toluene, 0.6 mmol) and 20 mL of toluene were added, the reaction mixture was stirred overnight under nitrogen at 110 °C for 24 h. The solution was then cooled to room temperature, followed by the addition of 50 mL water, which was extracted with 50 mL dichloromethane. The organic phases were combined and dried with anhydrous Na_2SO_4. The solution was filtered and the solvent was removed under vacuum to obtain the crude product. A red powder **1a** (0.103 g, 26%) and an orange powder **1b** (0.048 g, 12%) were obtained after isolation through silica column chromatography using petroleum ether and DCM (1:2, v/v) as the eluent (R_f = 0.45 for **1a**, 0.43 for **1b**). The characterization data of **1a**: IR (KBr disk), ν (cm^{-1}): 1654 (C=O), 1579, 1489, 1320, 1275, 783, 760, 699. ^1H NMR (500 MHz, $CDCl_3$) δ (TMS, ppm): 9.08 (d, J = 8.0 Hz, 1H), 8.95 (dd, J_1 = 4.6, J_2 = 1.9 Hz, 1H), 8.72 (dt, J_1 = 7.9, J_2 = 1.9 Hz, 2H), 8.41 (dd, J_1 = 8.4, J_2 = 1.2 Hz, 1H), 7.60 (t, J = 8.0 Hz, 1H), 7.51 (dd, J_1 = 7.9, J_2 = 2.7 Hz, 1H), 7.47 (dd, J_1 = 7.9, J_2 = 4.6 Hz, 1H), 7.28-7.20 (m, 4H), 7.10-7.04 (m, 4H), 7.02 (t, J = 7.0 Hz, 2H). ^{13}C NMR (125 MHz, $CDCl_3$) δ (TMS, ppm): 183.82 (C=O), 153.75, 153.06, 148.64, 148.54, 135.78, 132.52, 130.27, 130.25, 129.96, 129.42, 128.53, 126.97, 126.46, 125.97, 123.13, 122.91, 122.88. HRMS: m/z 398.1429 (M^+, calcd 398.1414). The characterization data of **1b**: IR (KBr disk), ν (cm^{-1}): 1647 (C=O), 1576, 1487, 1342, 1271, 771, 754, 696. ^1H NMR (500 MHz, $CDCl_3$) δ (TMS, ppm): 9.09 (d, J = 8.0 Hz, 1H), 8.96 (dd, J_1 = 4.6, J_2 = 1.9 Hz, 1H), 8.73 (dd, J_1 = 7.9, J_2 = 1.9 Hz, 1H), 8.69 (d, J = 8.4 Hz, 1H), 8.18 (dd, J_1 = 8.5, J_2 = 1.1 Hz, 1H), 7.54 (t, J = 8.0 Hz, 1H), 7.50 (dd, J_1 = 7.9, J_2 = 4.6 Hz, 1H), 7.45 (d, J = 8.1 Hz, 1H), 7.30-7.23 (m, 4H), 7.12-7.01 (m, 6H). ^{13}C NMR (125 MHz, $CDCl_3$) δ (TMS, ppm): 182.71(C=O), 153.43, 153.32, 152.27, 148.70, 135.64, 131.14, 130.58, 129.53, 129.00, 128.92, 127.57, 126.51, 126.47, 125.48, 124.72, 123.87, 123.51, 123.28. HRMS: m/z 398.1425 (M^+, calcd 398.1414).

Synthesis of 2a and 2b. Into a 100 mL two-necked round-bottom flask were added the mixture of **7** and **8** (0.31 g, 1.0 mmol), 4-(1,2,2-triphenylethenyl)-*N*-[4-(1,2,2-triphenylethenyl)phenyl]-benzenamine **10** (0.68 g, 1.0 mmol), Cs_2CO_3 (1.63 g, 5.0 mmol), and $Pd(OAc)_2$ (0.045 g, 0.2 mmol). The flask was vacuumed and purged with dry nitrogen for three times. After the solution of P(*t*-Bu)$_3$ (6 mL, 0.1 mol/L in toluene, 0.6 mmol) and 20 mL of toluene were added, the reaction mixture was stirred overnight under nitrogen at 110 °C for 24 h. The solution was then cooled to room temperature, followed by the addition of 50 mL water, which was extracted with 50 mL dichloromethane. The organic phases were combined and dried with anhydrous Na_2SO_4. The solution was filtered and the solvent was removed under vacuum to obtain the crude product. Red powders **2a** (0.110 g, 12%) and **2b** (0.115 g, 13%) were obtained after isolation through silica column chromatography using petroleum ether and DCM (1:2, v/v) as the eluent (R_f = 0.65 for **2a**, 0.62 for **2b**). The characterization data of **2a**: IR (KBr disk), ν (cm^{-1}): 1655 (C=O), 1575, 1500, 1315, 1278, 788, 758, 700. ^1H NMR (500 MHz, $CDCl_3$) δ (TMS, ppm): 9.02 (d, J = 8.0 Hz, 1H), 8.93 (dd, J_1 = 4.6, J_2 = 1.9 Hz, 1H), 8.73 (dd, J_1 = 7.3, J_2 = 1.2 Hz, 1H), 8.69 (dd, J_1 = 7.9, J_2 = 1.8 Hz, 1H), 8.28 (dd, J_1 = 8.4, J_2 = 1.2 Hz, 1H), 7.62 (t, J = 7.4 Hz, 1H), 7.45 (dd, J_1 = 7.9, J_2 = 4.6 Hz, 1H), 7.38 (d, J = 8.0 Hz, 1H), 7.18-6.95 (m, 30H), 6.86 (d, J = 8.7 Hz, 4H), 6.75 (d, J = 8.7 Hz, 4H). ^{13}C NMR (125 MHz, $CDCl_3$) δ (TMS, ppm): 183.87(C=O), 153.86,

153.14, 148.24, 146.58, 143.88, 143.62, 143.44, 140.75, 140.42, 138.38, 135.62, 132.58, 132.29, 131.34, 131.31, 130.19, 130.13, 129.63, 128.52, 128.13, 127.65, 127.61, 127.59, 126.55, 126.46, 126.45, 126.36, 126.04, 125.87, 122.85, 122.19. HRMS: m/z 906.3590 (M^+, calcd 906.3605). The characterization data of **2b**: IR (KBr disk), ν (cm^{-1}): 1645 (C=O), 1571, 1500, 1340, 1280, 773, 761, 700. ^1H NMR (500 MHz, $CDCl_3$) δ (TMS, ppm): 9.13 (d, J = 6.7 Hz, 1H), 8.97 (dd, J_1 = 4.5, J_2 = 1.9 Hz, 1H), 8.75 (d, J = 7.5, 1H), 8.65 (d, J = 8.1 Hz, 1H), 8.07 (d, J = 8.5 Hz, 1H), 7.56 (t, J = 7.5 Hz, 1H), 7.51 (dd, J_1 = 7.8, J_2 = 4.4 Hz, 1H), 7.32 (d, J = 8.1 Hz, 1H), 7.19-6.93 (m, 30H), 6.88 (d, J = 8.6 Hz, 4H), 6.74 (d, J = 8.6 Hz, 4H). ^{13}C NMR (125 MHz, $CDCl_3$) δ (TMS, ppm): 182.40(C=O), 153.05, 152.13, 146.56, 143.81, 143.55, 143.35, 141.01, 140.31, 139.18, 135.98, 132.41, 131.35, 131.30, 131.09, 130.57, 129.21, 128.61, 127.70, 127.63, 127.62, 126.60, 126.54, 126.52, 126.43, 126.08, 125.44, 124.40, 123.28, 122.97. HRMS: m/z 906.3617 (M^+, calcd 906.3605).

The preparation of nanoparticles:

The nanoparticles of **1a-b** and **2a-b** were prepared by nanoprecipitation. A solution was prepared by dissolving 1.0 mg of the dye and 2.0 mg of DSPE-PEG_{2000}-OCH_3 in 1 mL of $CHCl_3$, which was mixed with 10 mL of water in the presence of probe sonication with the input power of 12 W for 10 min. After that, nitrogen was purged into the solution to evaporate $CHCl_3$ to obtain a clear transparent aqueous suspension of nanoparticles with a dye concentration of 0.1 mg/mL.

ROS generation measurement:

The ROS generation measurements of **1a-b** and **2a-b** were conducted under the irradiation of a white LED lamp using 2,7-dichlorodihydrofluorescein (DCFH) as the indicator, which was generated from the reaction between DCFH-DA (0.5 mL, 1.0 mol/L) and aqueous solution of NaOH (2 mL, 1.0 mol/L) for 30 min in dark. 10 mL of PBS buffer solution was then added in the reaction mixture to obtain DCFH solution with a concentration of 40 mmol/L. To 2895 μL of PBS buffer solution was added 75 μL of DCFH solution (40 mmol/L). 30 μL of THF solution of the dye (1×10^{-3} mol/L) was then added under vigorous stirring to gain a mixture with the concentrations of the dye and DCFH to be 1×10^{-5} mol/L and 1×10^{-3} mol/L, respectively, whose PL spectra were collected before and after exposure to white LED lamp with different irradiation time. The irradiation input power was adjusted to 1.5 mW/cm^2 by changing the distance between the lamp and the solution. The fluorescence intensity at 525 nm was recorded to indicate the ROS generation rate. The same experimental procedure also applies to nanoparticles with the concentration of 1×10^{-5} M.

The determination of $^1O_2$ quantum yield:

Rose Bengal (RB) was used as the standard photosensitizer. The absorbance of RB (1×10^{-6} mol/L), nanoaggregates and nanoparticles of **1a-b** and **2a-b** with the effective concentration of 1×10^{-5} mol/L were tested. The integrations areas between 400–800 nm in the absorption spectra were calculated. The solution of photosensitizer with 2.5×10^{-6} mol/L of SOSG was exposed to the light from white Xe lamp with 7.0 mW/cm^2 input

power. The PL spectra were collected at different irradiation time and the fluorescence intensity at 525 nm was recorded to indicate the ROS generation rate. The fluorescence intensity at 525 nm *versus* the irradiation time gives a linear relationship, and the slope can be calculated as k_{RB} and k_{PS} in the presence of RB and PSs, respectively. The $^1O_2$ quantum yield of the photosensitizer in water (Φ_{PS}) was then calculated using the following formula:

$$\Phi_{PS} = \frac{\Phi_{RB} \times k_{PS} \int_{400nm}^{800nm} A_{RB}}{k_{RB} \int_{400nm}^{800nm} A_{PS}}$$

where A_{RB} and A_{PS} represent the light absorbance of RB and PSs, respectively; k_{RB} and k_{PS} are the increasing rate constants of fluorescence intensity of SOSG at 525 nm in the presence of RB and PSs, respectively. The $^1O_2$ quantum yield of RB (Φ_{RB}) is 0.75 in water.

Cell culture and bio-imaging experiments:
HeLa cells were cultured in DMEM medium containing 100 μg/mL streptomycin, 10% FBS, and 100 U/mL penicillin in a humidified incubator with 5% CO_2 at 37 °C in confocal imaging dishes. Into 995 μL of DMEM medium containing 100 μg/mL streptomycin, 10 vol% FBS, and 100 U/mL penicillin were added 5 μL of DMSO solution of **1a** (1 mmol/L) with/without 0.5 μL of DMSO solution of LysoTracker DND-26 (1 mmol/L) to gain a solution of **1a** with the concentration of 5 μmol/L in the presence/absence of 0.5 μmol/L of LysoTracker DND-26. The HeLa cells were then incubated with the mixture for 2 h in the dark, which were washed with PBS buffer solution (1.0 mL × 3) and imaged by a confocal microscope. For LysoTracker DND-26, the excitation wavelength was 488 nm and the emission filter was 500−540 nm (green); For **1a**, the excitation wavelength was 488 nm and the emission filter was 620−740 nm (red).

For the photostability evaluation, a selected area of confocal imaging was scanned continuously for 35 times with the scan rate of 7.75 s/scan by the CLSM laser light at 488 nm with the input power of 100%. The emission at 500-520 nm (DND-26) and 640-730 nm (**1a**) of the selected area are recorded.

For two-photon imaging experiments, the nanoparticles of **2a** were prepared by nanoprecipitation. A solution was prepared by dissolving 1.0 mg of **2a** and 12.0 mg of F127 in 2 mL of THF. The solvent was then removed under vacuum, followed by the addition of 1 mL of water to obtain an aqueous suspension of nanoparticles of **2a** with a concentration of 1 mg/mL. Nanoparticles of **2a** was injected into the mouse body through tail vein injection. The mouse brain vessels were then imaged using 1040 nm laser as excitation light and signal collected within 560-700 nm.

Cytotoxicity Studies:
MTT assays were used to assess the cell viability of Hela cells after incubation with **1a** without or with the exposure to the light from a white LED lamp (1.67 mW/cm^2) for different irradiation time. The cells in 96-well plates were incubated with **1a** for 2 h in

the dark to stain the cells with **1a**, which was then washed with PBS buffer solution and exposed to white light irradiation with different irradiation time. The cells were further incubated in fresh DMEM medium containing 100 μg/mL streptomycin, 10% FBS, and 100 U/mL penicillin in a humidified incubator with 5% CO_2 at 37 °C for 24 h and washed with PBS buffer solution. MTT solution (5 mg/mL in PBS buffer) was diluted by the same DMEM medium to gain the MTT working solution with the concentration of 0.5 mg/mL. 100 μL of MTT working solution was added into each well. After incubation for 3 h, the supernatant solution was discarded and the precipitate was dissolved in 100 μL of DMSO with gentle shaking. The absorbance of MTT at 570 nm was monitored by TECAN Infinite 200 PRO microplate reader. The cells without any treatment were also used as control.

The measurement of two-photon absorption cross section:

Rhodamine B (RhB) was used as the standard sample. The fluorescence intensity of RhB with concentration of 1×10^{-6} mol/L in methanol, nanoaggregates of **1a-b** and **2a-b** with the effective concentration of 1×10^{-5} mol/L in THF/H_2O mixtures with 99 vol% water were tested by 1040 nm laser as excitation light source at the same condition. Two-photon absorption cross section (σ) can be calculated using the following formula:

$$\frac{\sigma_1}{\sigma_2} = \frac{F_1 \cdot c_2 \cdot n_2 \cdot \Phi_2}{F_2 \cdot c_1 \cdot n_1 \cdot \Phi_1}$$

where F_1 and F_2 represent the fluorescence intensity of standard sample and tested sample, respectively; c_1, n_1, and φ_1 are the concentration, refractive index, and fluorescence quantum yield of RhB solution, respectively; c_2, n_2, and φ_2 are the concentration, refractive index, and fluorescence quantum yield of the tested sample, respectively. The two-photon absorption cross section of RhB (σ_{RhB}) is 39 GM in methanol with the fluorescence quantum yield of 0.6. The two-photon absorption cross sections of **1a** and **2a** in the aggregated states are measured to be 27 GM and 10 GM with 1040 nm excitation, respectively.

Cell Staining to distinguish live or dead cells:

The HeLa cells were cultured in DMEM medium containing 100 μg/mL streptomycin, 10% FBS, and 100 U/mL penicillin in a humidified incubator with 5% CO_2 at 37 °C in confocal imaging dishes. The cells were then incubated with **1a** (10 μmol/L) for 2 h in the dark, which were washed with 1 mL of PBS buffer solution and exposed to light irradiation (1.67 mW·cm^{-2}) for a certain time with the irradiation energy of 0–2.0 J·cm^{-2}. After light irradiation, the cells were cultured in DMEM medium containing 100 μg/mL streptomycin, 10% FBS and 100 U/mL penicillin in a humidified incubator with 5% CO_2 at 37 °C for 20 h. The cells were then washed with 1 mL of PBS buffer solution and incubated with fluorescein diacetate (50 μg/mL) and PI (50 μg/mL) for 10 min. After washing, the cells were imaged by CLSM after washing with 1 mL of PBS buffer solution. The green fluorescence from fluorescein diacetate is collected from 500 to 525 nm upon excitation at 488 nm, and the red fluorescence from PI is collected from 600 to 630 nm upon excitation at 543 nm.

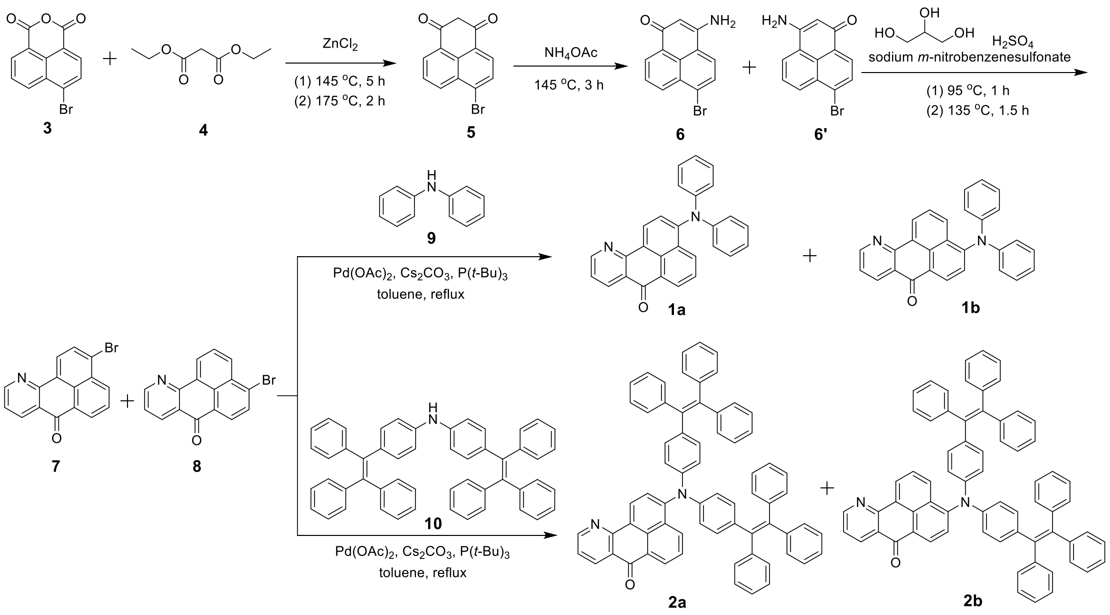

Scheme S1. Synthesis routes of **1a-b** and **2a-b**.

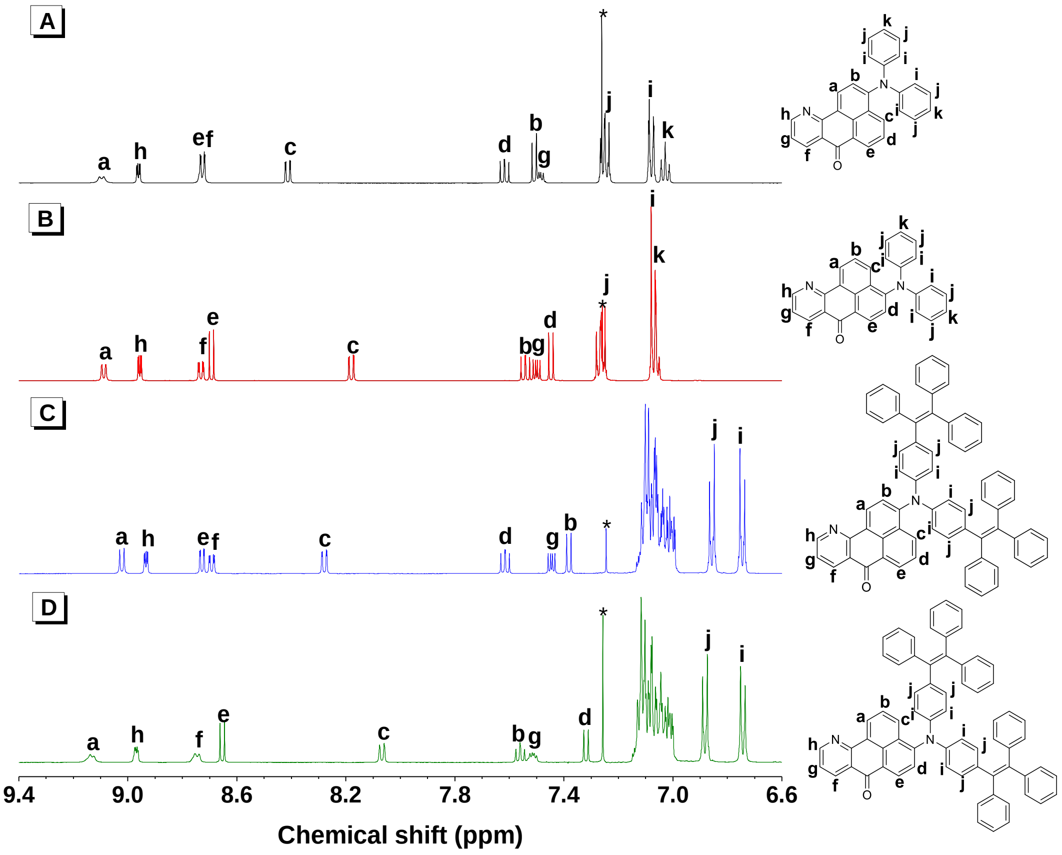

Fig. S1 ^{1}H NMR spectra of (A) **1a**, (B) **1b**, (C) **2a**, and (D) **2b** in $CDCl_3$. The solvent peaks are marked with asterisks.

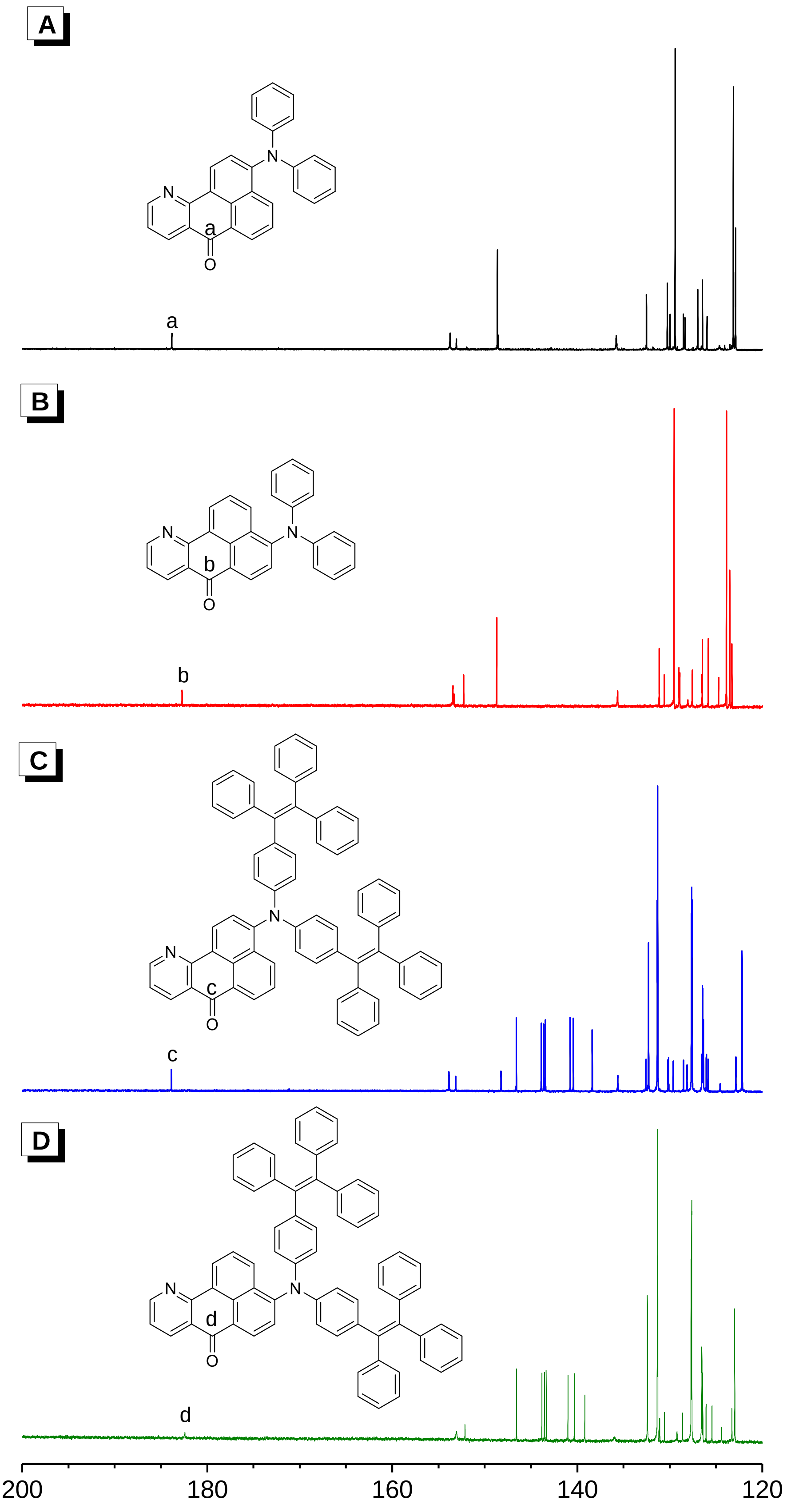

Fig. S2 ^{13}C NMR spectra of (A) **1a**, (B) **1b**, (C) **2a**, and (D) **2b** in $CDCl_3$.

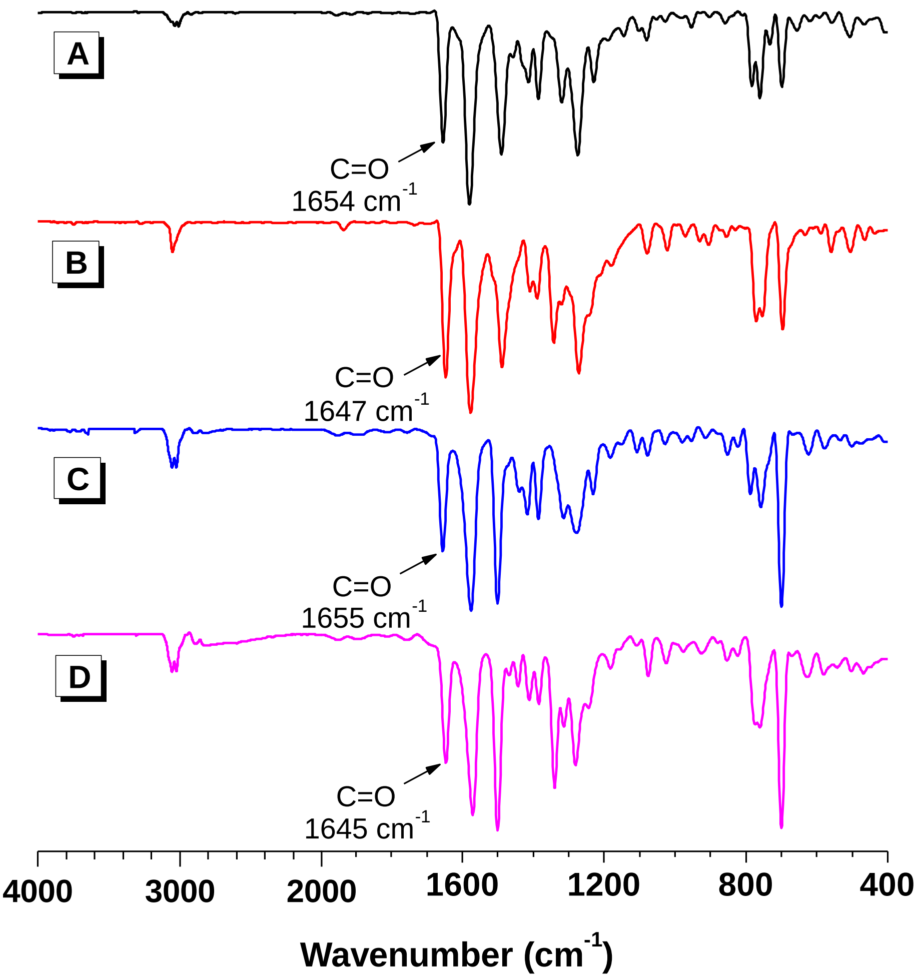

Fig. S3 IR spectra of (A) **1a**, (B) **1b**, (C) **2a**, and (D) **2b**.

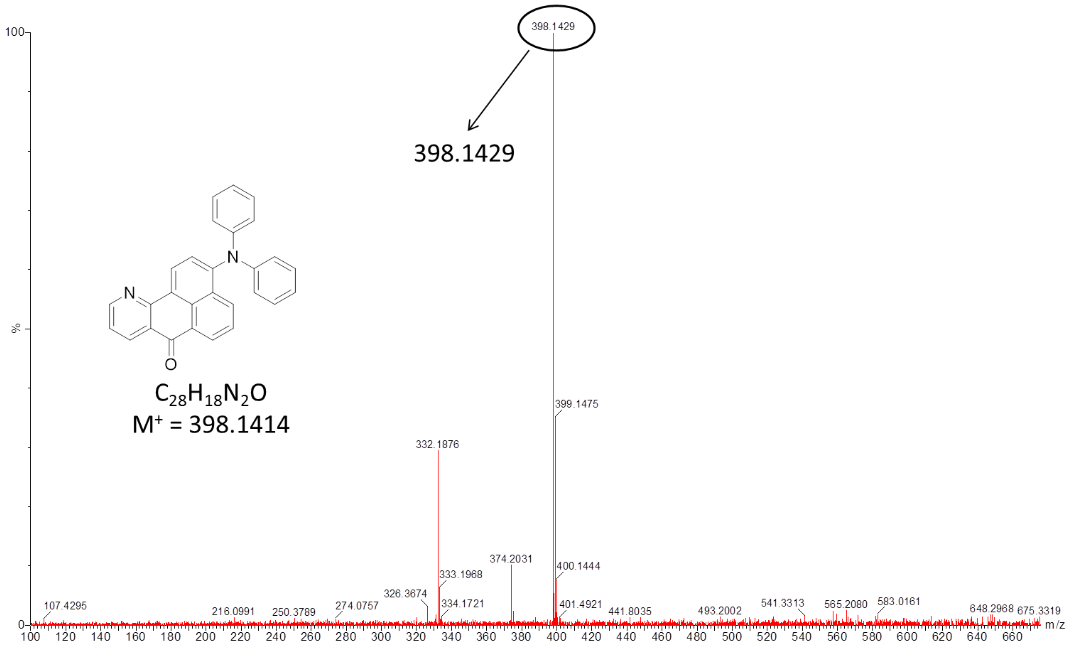

Fig. S4 HR-MS spectrum of **1a**.

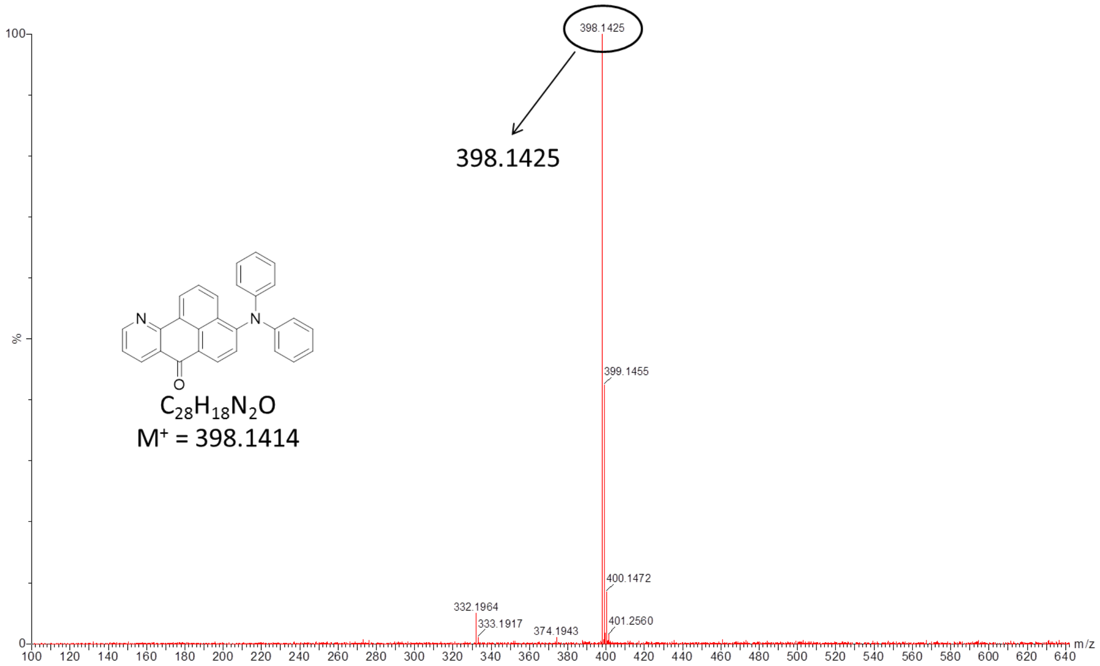

Fig. S5 HR-MS spectrum of **1b**.

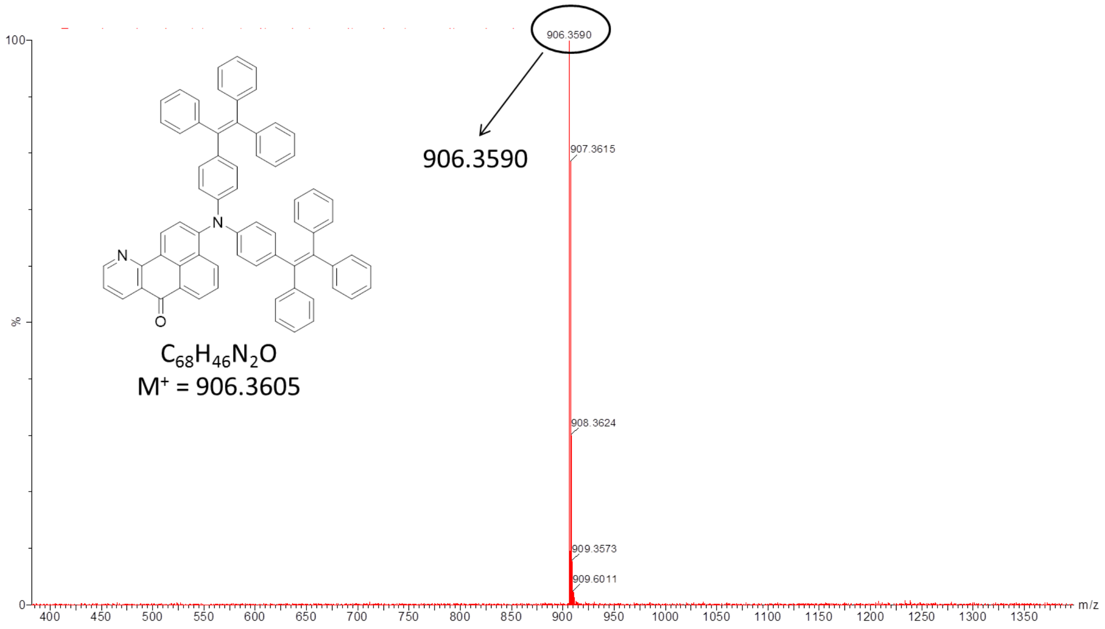

Fig. S6 HR-MS spectrum of **2a**.

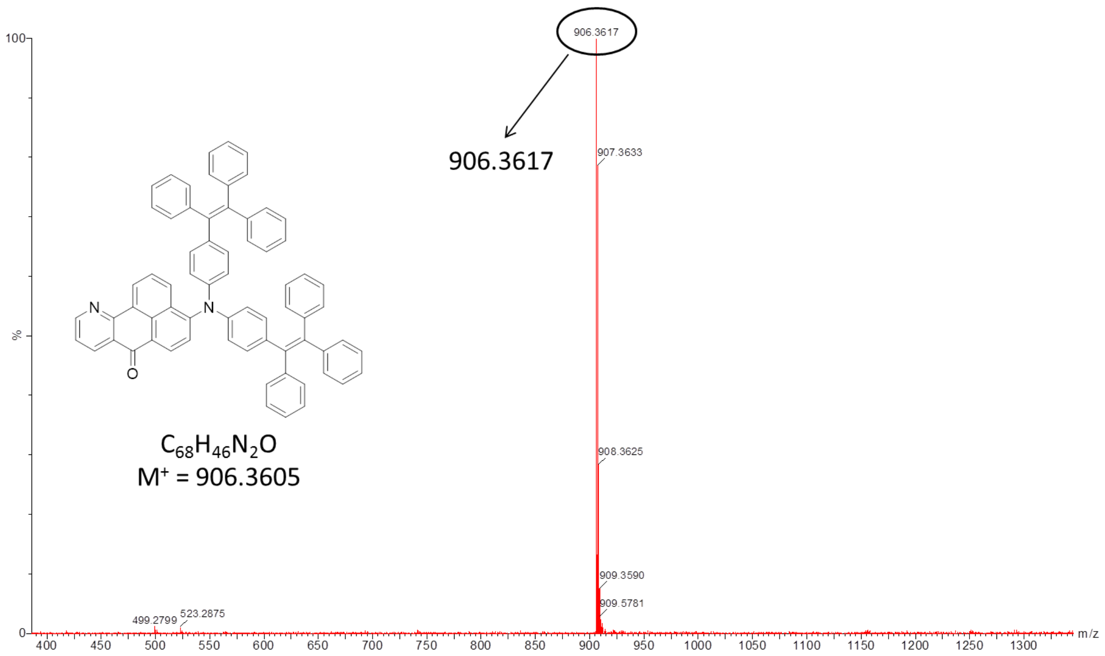

Fig. S7 HR-MS spectrum of **2b**.

Table S1. Crystal data and structure refinement for single crystals **1a-b**.

Crystal form	**1a**	**1b**
CCDC number	1822247	1822250
Empirical formula	$C_{28}H_{18}N_2O$	$C_{28}H_{18}N_2O$
Formula weight	398.44	398.44
Temperature (K)	293	293
Wavelength (Å)	0.71073	0.71073
Crystal system, space group	monoclinic, P 1 21/c 1	triclinic, P-1
	a = 7.4630(14) Å	a = 7.1516(4) Å
	b = 23.830 (5) Å	b = 8.5555(5) Å
Unit cell dimensions	c = 11.556(3) Å	c = 16.6112(10) Å
	α = 90°	α = 100.052(5)°
	β = 101.301(18)°	β = 95.016(5)°
	γ = 90°	γ = 90.498(5)°
Volume (Å^3)	2015.3 (8)	996.63(10)
Z, Calculated density (g cm^{-3})	4, 1.313	2, 1.328
Absorption coefficient (mm^{-1})	0.080	0.081
F(000)	832.0	416.0
Theta max for data collection	25.347°	25.350°
	h <= 8	h <= 8
Limiting indices	k <= 28	k <= 10
	l <= 13	l <= 20
Reflections collected / unique	5511 / 3662	3643/ 3655
Completeness to theta	1.505	0.997

Max. and min. transmission	1.000 and 0.771	1.000 and 0.685
Refinement method	Full-matrix least-squares on F^2	
Data / parameters	2397 / 281	3075 / 280
R indices (all data)	R1 = 0.0688, wR2 = 0.2031	R1 = 0.0549, wR2 = 0.1659

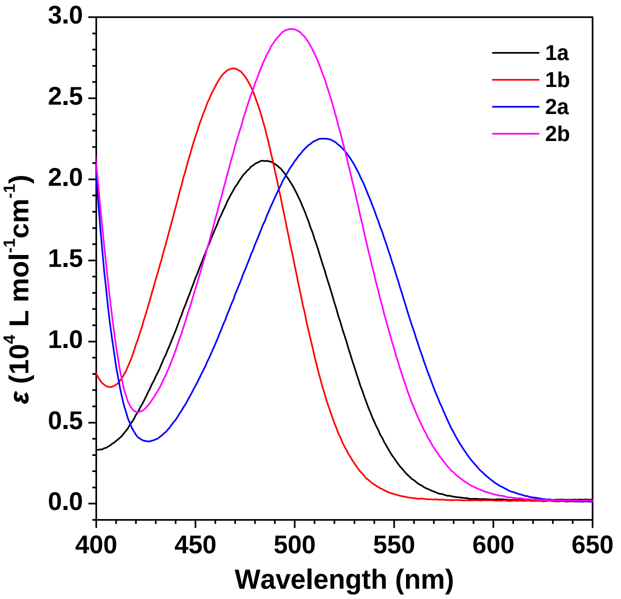

Fig. S8 UV-Vis absorption spectra of THF solutions of **1a**-**b** and **2a**-**b**. Concentration: 10 μM.

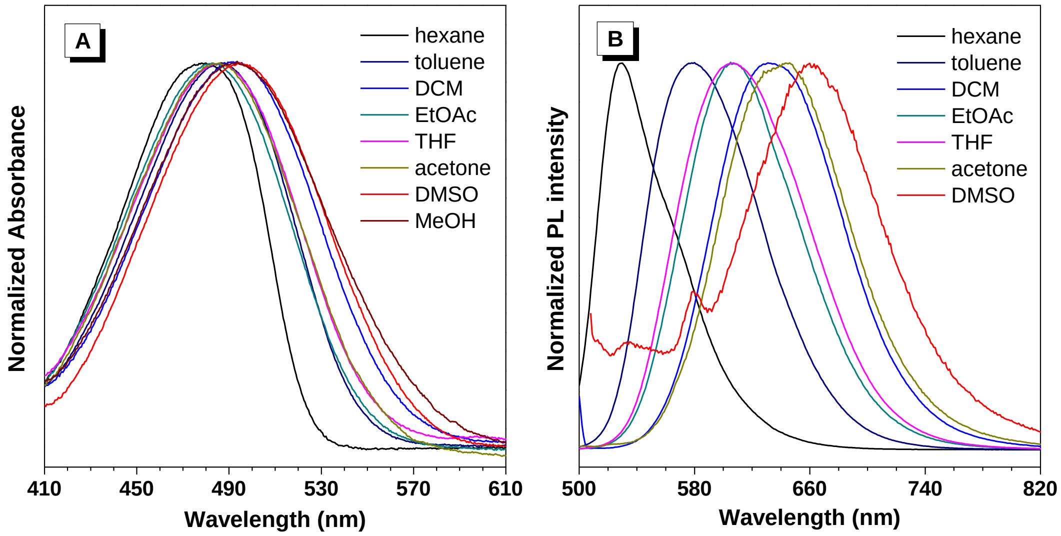

Fig. S9 Normalized (A) UV-Vis absorption spectra and (B) PL spectra of **1a** in different solvents. Concentration: 10 μM.

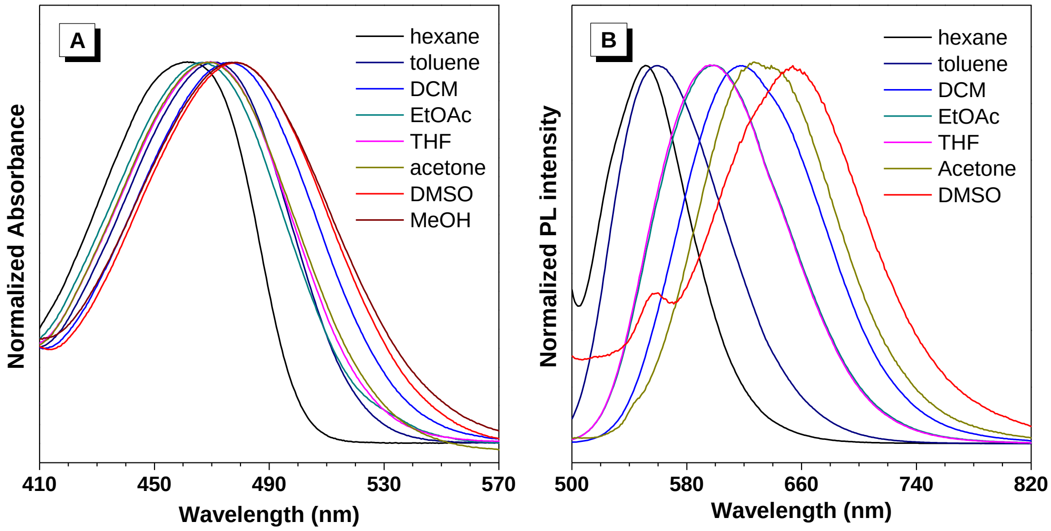

Fig. S10 Normalized (A) UV-Vis absorption spectra and (B) PL spectra of **1b** in different solvents. Concentration: 10 μM.

Table S2. Photophysical properties of **1a-b** in different solvents.

solvent	**1a**					**1b**				
	λ_{ab} (nm)	ε (L·mol^{-1}·cm^{-1})	λ_{em} (nm)^{a}	τ (ns)^{b}	Φ_f^{c}	λ_{ab} (nm)	ε (L·mol^{-1}·cm^{-1})	λ_{em} (nm)^{a}	τ (ns)^{b}	Φ_f^{c}
hexane	480	24100	529	11.5	0.83	462	30450	552	5.7	0.36
toluene	487	21960	579	11.7	0.69	471	29990	560	9.1	0.45
DCM	493	20470	633	7.0	0.23	476	24600	619	4.8	0.13
EtOAc	483	23240	607	8.7	0.32	467	27470	598	5.7	0.14
THF	486	21300	607	9.8	0.39	469	26930	598	7.1	0.21
acetone	486	21660	642	2.4	0.06	469	24470	634	1.7	0.03
DMSO	495	18930	662	0.9	0.01	478	23680	654	0.9	0.007
MeOH	494	17880	n.d.	n.d.	n.d.	478	26840	n.d	n.d.	n.d.

$^{a}\lambda_{ex}$ = λ_{abs}. $^{b}\lambda_{ex}$ = 470 nm. ^{c}absolute PL quantum yield determined with a calibrated integrating sphere system and λ_{ex} = λ_{ab}. n.d. = not detectable. Concentration: 10 μM.

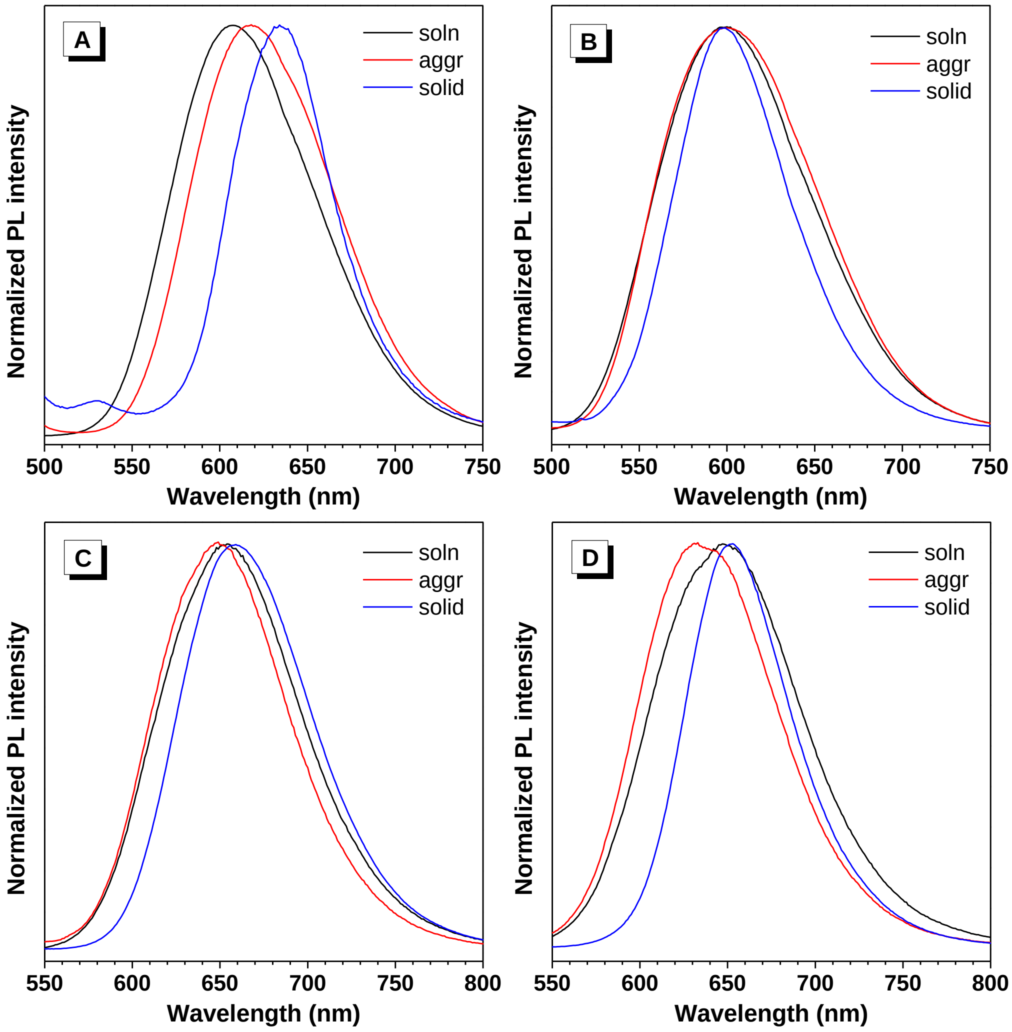

Fig. S11 Normalized PL spectra of (A) **1a**, (B) **1b**, (C) **2a**, and (D) **2b** in solution, aggregated and solid states. soln: in THF solution (10 μM); aggr: in THF/water mixture with 99 vol% water contents (10 μM); solid: solid powder of the compounds.

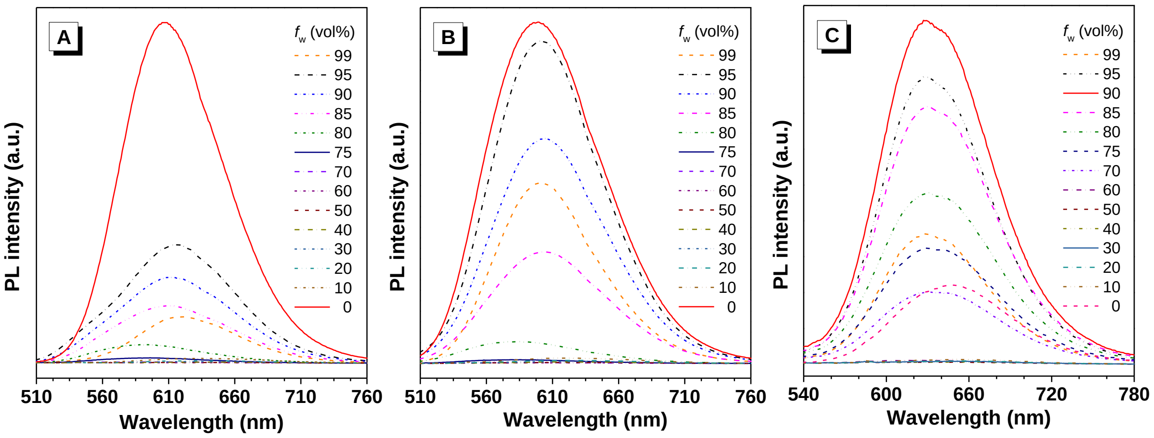

Fig. S12 PL spectra of (A) **1a**, (B) **1b**, and (C) **2b** in THF/water mixtures with different fractions of water (0-99 vol%), $\lambda_{ex} = \lambda_{ab}$, concentration: 10 μM.

Table S3. Particle size measurements of nanoaggregates and nanoparticles of **1a-b** and **2a-b** by dynamic light scattering technique[a]

sample	diameter (nm)	sample	diameter (nm)
**1a** aggr	150	**1a** NP	140
**1b** aggr	120	**1b** NP	130
**2a** aggr	160	**2a** NP	140
**2b** aggr	170	**2b** NP	120

[a]aggr: nanoaggregates formed in THF/water mixture with 99 vol% water content, concentration = 10 μmol/L; NP: nanoparticle prepared by nanoprecipitation. All the data were retest for 3 times to gain the average diameter of particles.

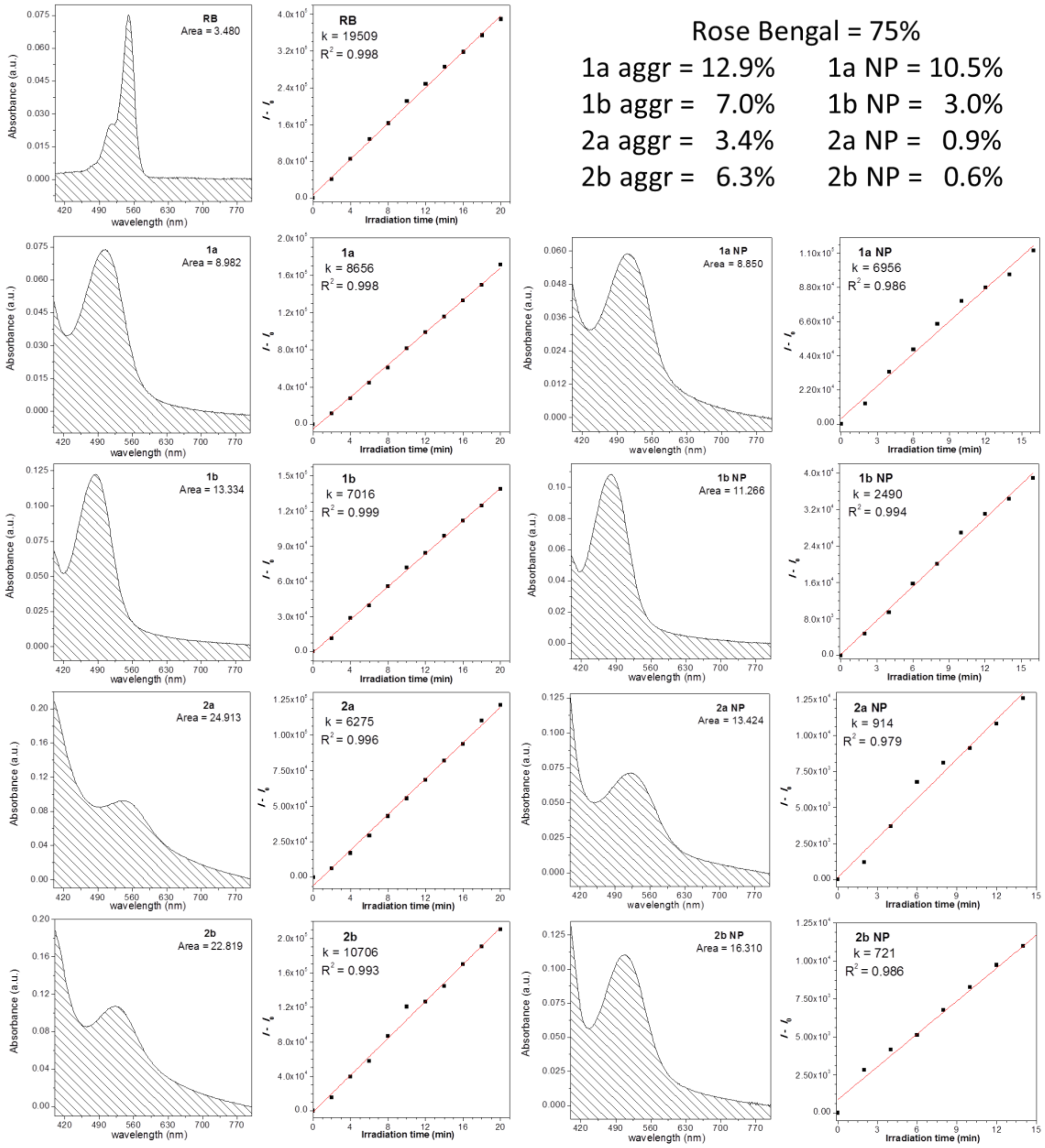

Fig. S13 The measurement of $^1O_2$ quantum yield of nanoaggregates and nanoparticles of **1a-b** and **2a-b** using SOSG as indicator.

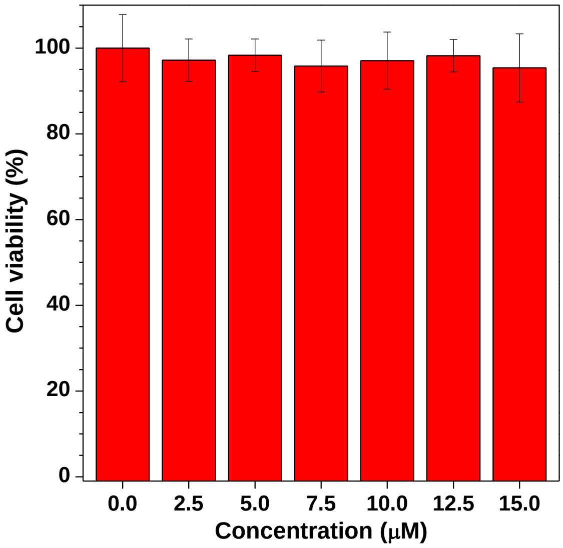

Fig. S14 Cytotoxicity of **1a** for Hela cells without light irradiation. Cell viabilities of Hela cells were tested by MTT method.

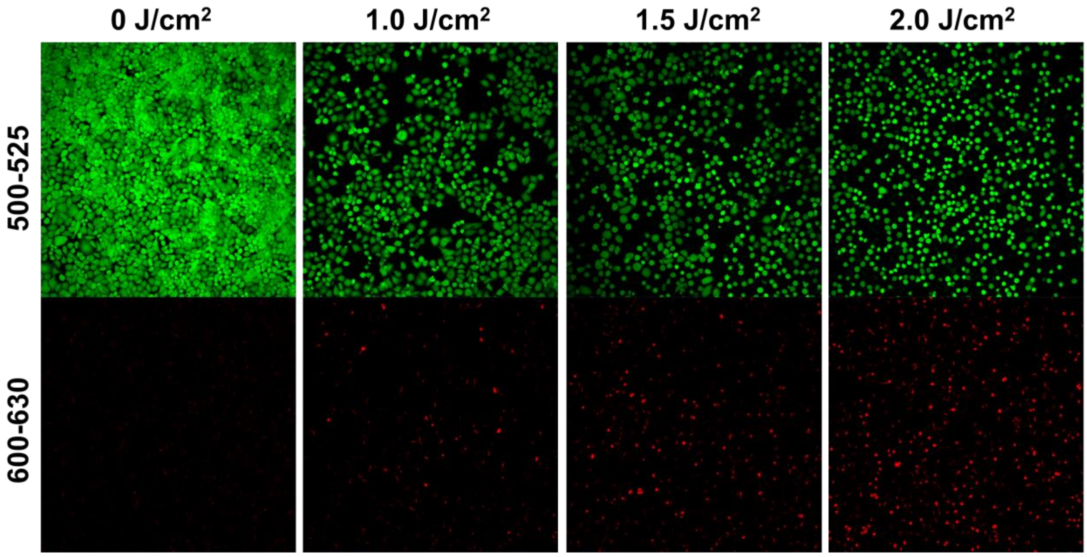

Fig. S15 Cell imaging with fluorescein and PI for HeLa cells incubated with **1a** for 2 h in the dark and exposed to white light irradiation (1.67 mW·cm^{-2}) with different irradiation energy. The living cells were stained by fluorescein diacetate (green, 500–525 nm channel, 50 μg/mL for 10 min) and the dead cells were stained by PI (red, 600–630 nm channel, 50 μg/mL for 10 min).
